# Dynamic Modelling, Process Control, and Monitoring of Selected Biological and Advanced Oxidation Processes for Wastewater Treatment: A Review of Recent Developments

**DOI:** 10.3390/bioengineering11020189

**Published:** 2024-02-16

**Authors:** Zahra Parsa, Ramdhane Dhib, Mehrab Mehrvar

**Affiliations:** Department of Chemical Engineering, Toronto Metropolitan University, 350 Victoria Street, Toronto, ON M5B 2K3, Canada; zahra.parsa@torontomu.ca (Z.P.); rdhib@torontomu.ca (R.D.)

**Keywords:** process control, dynamic modelling, online monitoring, real-time monitoring, wastewater treatment, biological wastewater treatment, ASPs, SBRs, AOPs

## Abstract

This review emphasizes the significance of formulating control strategies for biological and advanced oxidation process (AOP)-based wastewater treatment systems. The aim is to guarantee that the effluent quality continuously aligns with environmental regulations while operating costs are minimized. It highlights the significance of understanding the dynamic behaviour of the process in developing effective control schemes. The most common process control strategies in wastewater treatment plants (WWTPs) are explained and listed. It is emphasized that the proper control scheme should be selected based on the process dynamic behaviour and control goal. This study further discusses the challenges associated with the control of wastewater treatment processes, including inadequacies in developed models, the limitations of most control strategies to the simulation stage, the imperative requirement for real-time data, and the financial and technical intricacies associated with implementing advanced controller hardware. It is discussed that the necessity of the availability of real-time data to achieve reliable control can be achieved by implementing proper, accurate hardware sensors in suitable locations of the process or by developing and implementing soft sensors. This study recommends further investigation on available actuators and the criteria for choosing the most appropriate one to achieve robust and reliable control in WWTPs, especially for biological and AOP-based treatment approaches.

## 1. Introduction

Despite the finite water resources on Earth, the demand for water is continuously increasing. Therefore, wastewater treatment is necessary to clean and recycle used water for consumption. Indeed, the objective of wastewater treatment plants (WWTPs) is not to produce a profit-making product but to protect water as an asset. Additionally, releasing untreated or inadequately treated wastewater into the environment poses risks such as eutrophication, the release of toxic substances, heavy metals, and other harmful materials, endangering the ecosystem. As a result, there has been a notable trend towards establishing closed-loop wastewater treatment systems in recent years. These systems aim to reintegrate treated water into the consumption cycle while maximizing the recycling and recovery of nutrients, metals, and energy [1,2,3,4,5,6].

**Figure 1:**
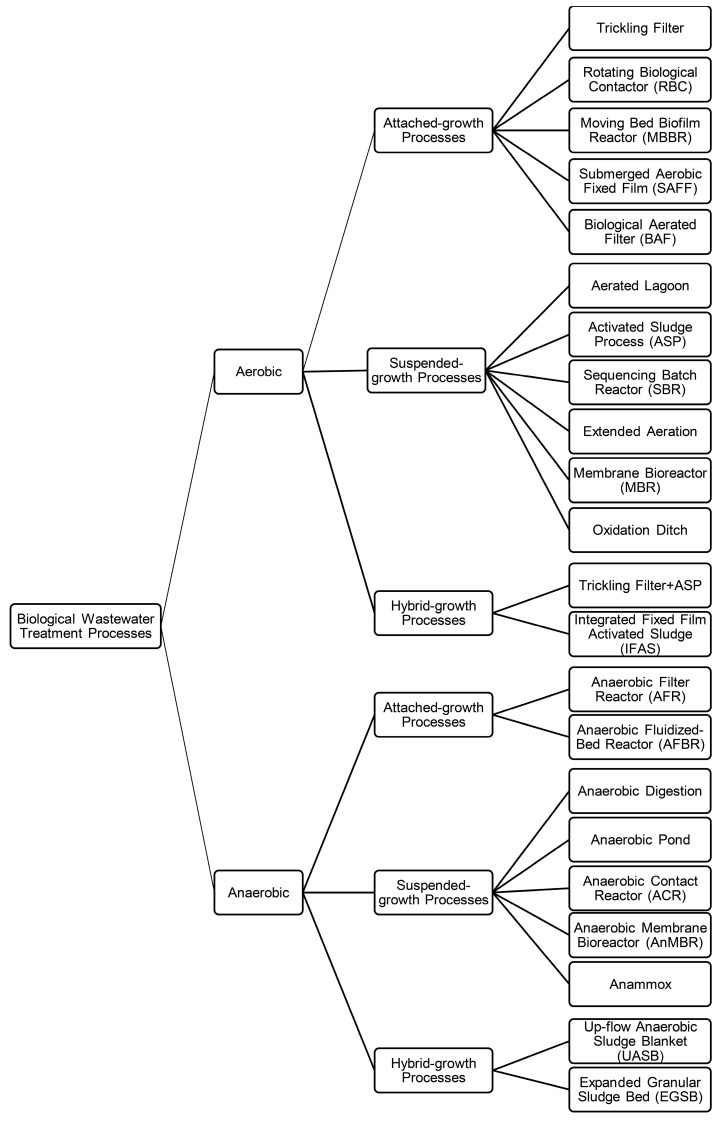
Classification of major biological wastewater treatment processes (adapted from [7,8,9]).

The primary objective of municipal wastewater treatment plants (MWWTPs) is to degrade organics and nutrients. On the contrary, treating industrial wastewater is more challenging due to its varying characteristics depending on the industry type. In MWWTPs, the predominant organic degradation occurs in the biological treatment stage. Figure 1 illustrates the categorization of major biological treatment methods into aerobic and anaerobic processes [7,8,9]. Each of these processes is further classified as suspended-growth, attached-growth, or hybrid-growth, depending on the dominant mechanism whereby microorganisms are incorporated into the treatment process. Aerobic biological treatment processes generally dominate wastewater treatment in both MWWTPs and industrial wastewater treatment plants (IWWTPs). However, aerobic methods may prove less effective in cases of exceptionally high organic content, prompting consideration of anaerobic processes as the preferred biological treatment approach [10]. Commonly adopted biological processes in WWTPs are suspended-growth, among which the most frequently observed aerobic suspended-growth biological processes, especially in MWWTPs, are the activated sludge process (ASP) and the sequencing batch reactor (SBR) [11,12,13]. While biological wastewater treatment is favoured for its economic advantages and generally effective performance, its efficacy diminishes when addressing nonbiodegradable, recalcitrant, and high molecular weight compounds. In recent decades, the concentrations of these compounds have increased in urban and industrial wastewater, due to their high usage in manufacturing and presence in final products [14].

**Figure 2:**
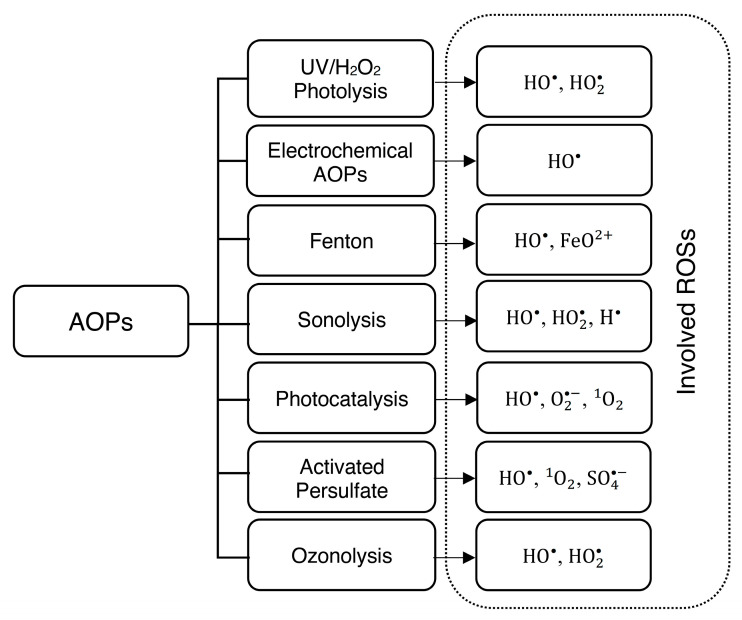
Different AOPs involving ROS (adapted from [15]).

Advanced oxidation processes (AOPs) have demonstrated notable performance in degrading various organics, including refractory ones. These processes primarily rely on the non-selective reaction of in situ-produced hydroxyl radicals (HO^•^) and other reactive oxygen species (ROS) with organic contaminants. The reaction rates are typically significant, ranging between 10^8^ and 10^11^ $M^{-1}s^{-1}$ [15,16]. As a result, these mechanisms have the potential to oxidize various contaminants including low-concentration, toxic, or nonbiodegradable organics [17,18]. Some of the common AOPs with involved ROS are shown in Figure 2. It is important to note that when compounds are degraded in certain AOPs, complex by-products may be generated. Due to the strong atomic bonds in their molecules, such by-products may resist further degradation, subsequently hindering mineralization [19,20]. This challenge is also encountered in some real WWTPs [21]. However, some studies affirm the effectiveness of AOPs in diminishing chronic daily intake (CDI) and hazard quotient (HQ) linked to specific recalcitrant pollutants [22,23]. Additionally, numerous studies have proven that AOPs enhance the biodegradability of low-biodegradability wastewater [14,22,23,24,25] and produce low-toxicity, biodegradable by-products compared to the original pollutants. In some cases, even the complete mineralization of contaminants has been reported [25]. Therefore, contemplating the utilization of AOPs as a viable strategy for addressing recalcitrant pollutants merits consideration if the preliminary lab-scale experimental assessment has been performed.

**Figure 3:**
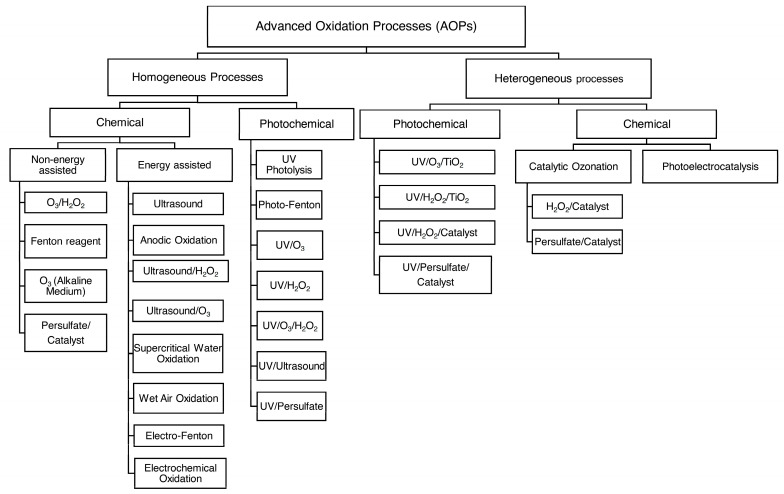
Classification of different AOPs (adapted from [26]).

AOPs can be classified into homogeneous and heterogeneous reactions based on the number of phases in the oxidation reaction [26]. As depicted in Figure 3, each of these classes is divided into chemical and photochemical processes based on whether light is involved in the process or not. Additionally, beyond the conventional AOPs outlined in Figure 3, high-energy AOPs such as electron beam (EB) and non-thermal plasma (NTP) have shown significant efficiency in removing specific pollutants. Their application is particularly notable when conventional AOPs cannot achieve optimal mineralization [27].

Despite the efficiency of AOPs in removing refractory pollutants, their implementation in full-scale applications is challenging due to the high operating costs and the need for continuous monitoring to ensure the quality of the effluent. Optimizing AOP processes and implementing adequate controls for them can result in maintaining treated effluent quality within acceptable regulatory ranges while reducing operating costs.

Over the past few decades, numerous studies have aimed to enhance the efficacy of water and wastewater treatment technologies. These efforts involve implementing novel treatment methods and conducting optimization studies on both traditional and innovative approaches. Although these studies have improved the efficiency of treatment processes, a significant number of them are limited to study processes at their steady-state conditions. Nevertheless, most real processes do not keep operating at steady-state conditions. In other words, operational conditions change over time due to unexpected disturbances and uncertainties. These uncertainties encompass various factors. One factor involves disturbances in the ambient conditions. Another factor is the fluctuation in process inputs, such as variations in influent flow rates resulting from seasonal weather changes [28]. Additionally, there might be abrupt variations in influent characteristics due to the introduction of unexpected chemicals into wastewater, often discharged by industrial sectors into the sewer [29]. Furthermore, the complex nonlinear behaviour of processes, exemplified by the ASP under different operating conditions, adds to the array of uncertainties. Sometimes, the uncertainty is due to the high difference between the actual process variables and their predicted values by the developed steady-state model [30]. The dynamic behaviour of the process can be modelled and understood by studying the changes in process output(s) over time resulting from applying known changes to the process input variable(s).

Knowing the process dynamic behaviour, as the first crucial step in designing an efficient control, helps to anticipate the system output changes due to disturbances or changes in process inputs. This knowledge can be used in a feedforward (FF) or model predictive control (MPC) to prevent undesired violations in output or a feedback (FB) control to regulate the process by manipulating the manipulated variable (MV), after indicating offsets. Consequently, such control systems aid WWTPs in regulating the process and maintaining the effluent quality at desired discharge values to meet environmental discharge regulations or potable water standards [31,32]. Minimizing costs and maintaining process safety are other common, desirable process control goals.

The challenges in controlling biological wastewater processes stem from their inherent complexity. This complexity is evident in the intricate dynamic responses of microorganisms to elevated concentrations of unconventional pollutants. Additionally, variable process time constants and fluctuations in influent characteristics and flow rates contribute to these challenges. In WWTPs, the setpoints of controlled variables (CVs) are not constant over time due to the changes in regulations, weather, and influent conditions. In these scenarios, sophisticated control techniques can effectively regulate the process by adapting to the new setpoint [33,34].

In a control scheme, regulating the process to reject disturbance or track the reference trajectory can be carried out manually (open-loop system) or automatically (closed-loop system). Automation reduces the need for human intervention and results in decreasing operational costs. Also, a reliable and precise automatic system can maintain desired effluent quality and process safety by fast and immediate responses to process deviation from the desired target. Therefore, considering strict environmental discharge standards and the importance of providing healthy drinking water for consumers, automation in water treatment plants and WWTPs is highly beneficial and advantageous when properly implemented. The real-time monitoring is critical to provide fast and reliable control for WWTPs, avoiding releasing insufficiently treated water into the environment [35].

Therefore, this study aims to provide readers with a summary of dynamic modelling, process control, and monitoring of selected biological and AOP-based wastewater treatment processes. Considering the prevalence of the ASP and the SBR as the predominant aerobic biological processes in WWTPs, these two processes were selected to outline their modelling, process control, and monitoring.

## 2. System Identification/Modelling

**Figure 4:**
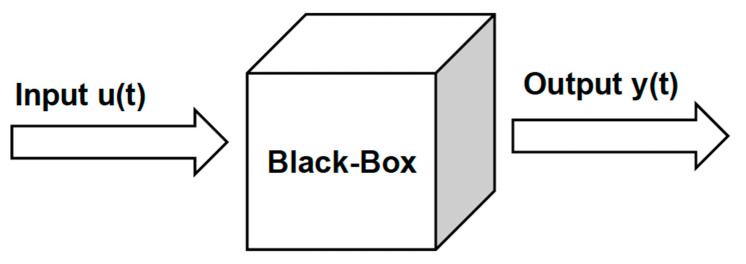
Black-box system identification diagram for capturing the dynamic behaviour of a system. The known input, u(t), can be sinusoidal, pulse, step, or pseudo-random binary sequence (PRBS), resulting in process output y(t) as frequency response, pulse response, or process reaction curve.

Depending on process complexity, a dynamic model is developed using either mechanistic models, which rely on kinetics, chemical, and physical information, or through system identification using experimental data. Subsequently, black-box system identification, as illustrated in Figure 4, or data-driven models (DDM) are exclusively derived from input–output experimental data.

**Figure 5:**
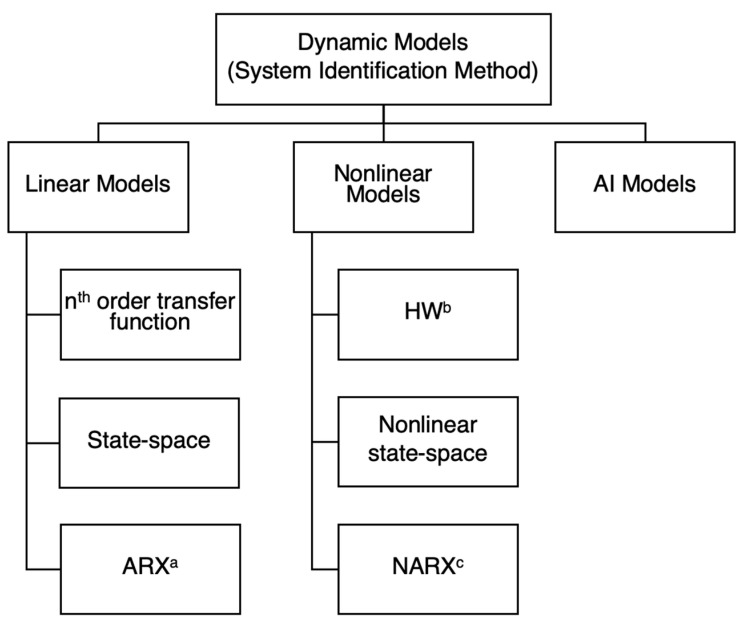
Different model structures of system identification: ^a^ autoregressive with eXogenous input model; ^b^ Hammerstein–Wiener model; ^c^ non-linear AutoRegressive with eXogenous input model (adapted from [37,38]).

Figure 5 shows different model structures of system identification, employed to map dynamic processes. Dynamic models are classified as linear, nonlinear, and artificial intelligence (AI)-based models constructed from an optimization scheme. In fact, to estimate model parameters, the weighted quadratic norm of the prediction error, $V_{N}\left(\theta ,N\right)$, must be minimized. The expression for $V_{N}\left(\theta ,N\right)$ is given by the following equation:

(1) $$V_{N}\left(\theta ,N\right)=\frac{1}{N}\sum_{k=1}^{N}\varepsilon^{2}(k,\theta )$$

where ε(k, θ) represents the difference between the actual process output ($y_{m}\left(k\right)$) and the predicted process output (y(k,θ)), with N being the number of samples in the training data set.

(2) $$\varepsilon \left(k,\theta \right)=y_{m}\left(k\right)-y(k,\theta )$$

**Figure 6:**
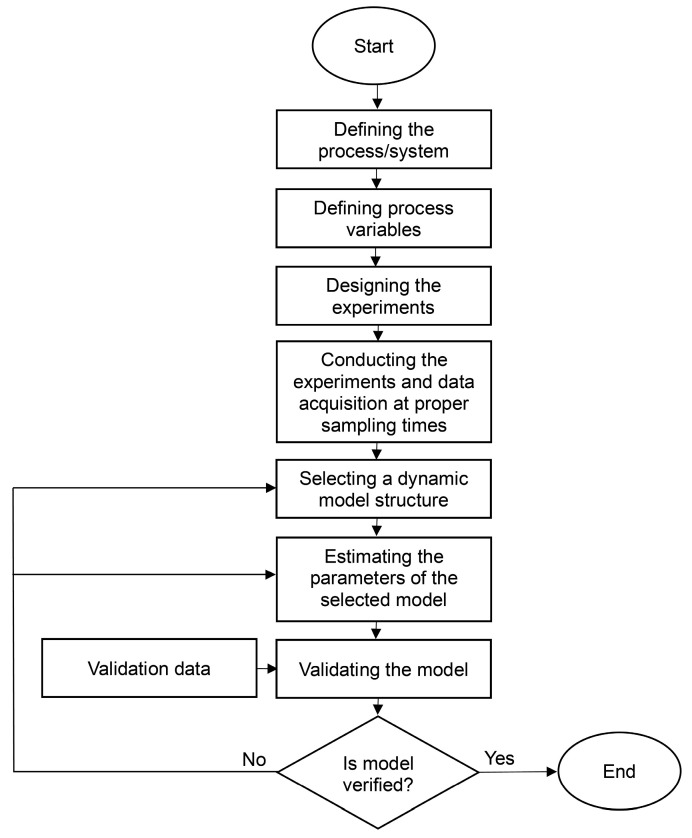
General flowchart of system identification technique.

As the complexity of a system increases, mapping its dynamic behaviour using linear structures is less accurate. Hence, implementing nonlinear structures, including AI-based structures, results in a dynamic model with a better fit. The parameters of each dynamic structure, illustrated in Figure 5, are estimated based on the basic mathematical equation presented earlier. Describing each modelling structure is out of the scope of this study and has been discussed in our previous study [36,37]. Figure 6 illustrates the necessary steps prior to, during, and after the system identification.

System identification is acknowledged as a valuable tool to elucidate the dynamic behaviour of complex and nonlinear water/wastewater treatment processes, especially biological and chemical ones such as ASPs, SBRs, and AOPs.

### 2.1. Modelling Biological Treatment Processes—ASPs

The widespread implementation of biological treatment processes in WWTPs motivates researchers to delve deeper for enhanced process understanding [39]. Further, the knowledge gained of the degradation is used to optimize the wastewater treatment process by increasing the degradation efficiency and decreasing costs. In addition, a control system must be applied to keep the process operating at its optimal conditions by designing a suitable control strategy. Also, it must be considered that the ASP is a nonlinear and complex process [39]. The dynamic complexity of ASPs arises primarily from the dynamic variations in microorganisms’ behaviour when exposed to disturbances. This complexity is further compounded by the fact that the ASP integrates various biological and physical processes [40]. Biological processes exhibit slower kinetics with a relatively larger average hydraulic retention time (HRT) than chemical processes. The large HRT contributes to a significant time delay, representing the lag between process input changes and corresponding output responses in the control system.

Due to the significance of the ASP in wastewater treatment, many studies have been conducted on developing activated sludge models (ASMs). As a result, standard mathematical models, including ASM1, ASM2, ASM2d, ASM3, ASM4, and ASM7, shown in Table 1, have been developed by the International Water Association (IWA) to describe and predict the biological behaviour of ASPs [12,41]. These models provide valuable information for the design, operation, and control of WWTPs, helping to optimize their performance and ensuring effective operations. Each model has a specific application and provides a different level of detail for the treatment process.

In conclusion, selecting the proper model to use depends on specific requirements in a particular WWTP. Implementing these general models into a WWTP requires calibration, which entails adjusting the model coefficients based on plant-specific data. Sometimes, designing and implementing an effective process control system based on these models is impractical due to their complex structure. Consequently, some studies have employed reduced, modified, altered, or simplified ASMs for process control [41]. For example, Smida et al. [41] developed a cascade high gain observer (HGO) to approximate the unknown input values (UI) and unknown input state variables (UIS) of a reduced ASM for an ASP by measuring only two state variables. The two state variables were dissolved oxygen (DO) and ${NO}_{3}^{-}$ concentrations. In their study, UIs were inlet ammonia and inlet substrate concentrations, whereas the UISs were the concentration of biodegradable substrate and the concentration of ammonia.

In addition to standard ASMs, two benchmark simulation models (BSMs), including BSM1 and BSM2, have been developed to evaluate the design and control strategies for ASPs [34,42]. In fact, BSMs serve as benchmark models to compare and assess the performance of different treatment system designs and proposed controls, while ASMs provide a more detailed and comprehensive description of the biological and chemical processes in WWTPs. Moreover, specialized software applications, such as SIMBA, GPS-X, BioWin, and WEST, incorporating dynamic models for ASPs, provide valuable assistance in analyzing and simulating wastewater treatment systems [43].

Although ASMs have shown good performance, they contain too many differential equations requiring specific data to be calibrated. As a result, some studies have been investigated employing other modelling methods to predict some parameters based on monitoring alternative process parameters. For instance, to find the dynamic relationship of the suspended solids (SS) content in the effluent of a WWTP with its influent characteristics, a fuzzy partial least square-based–dynamic Bayesian network (FPLS-DBN) has been employed [44]. This model is a combination of the Bayesian network (BN) with the fuzzy partial least squares (FPLS) methods. It has been observed that the developed approach can capture the nonlinearity and complexity of the process and predict the process output better than other conventional modelling methods and better than the BN or the FPLS alone [44]. Also, in another study, monitored data from the ASP in a WWTP were used to estimate the parameters of the state-space model representing the ASP [45]. Sadeghassadi et al. [46] used the autoregressive with exogenous input model (ARX) structure to model the ASP, and the results demonstrated a notable alignment between the actual and predicted data using the developed model. Novotny et al. [47] formulated a transfer function (TF) utilizing an autoregressive moving average stochastic model (ARMA). The TF aimed to describe the dynamic linear interdependency of mixed liquor suspended solids (MLSS) within the ASP and other pertinent process parameters. The data used for this formulation were collected from the Green Bay WWTP, Wisconsin. A noteworthy aspect of this derived model lies in its conformity with the mass balance model of the process. This alignment rendered the developed model mechanistic in nature rather than a black-box identification. Furthermore, in the same study, an artificial neural network (ANN) was utilized as a black-box model to predict the nonlinear correlation between the number of *filamentous* microorganisms in the ASP of the Jones Island WWTP, Milwaukee, WI, and various process parameters over time.

### 2.2. Modelling Biological Treatment Processes—SBR

The SBR process in a WWTP operates in a cyclic manner to treat continuously incoming wastewater. The choice between the ASP (also known as conventional activated sludge (CAS)) or SBR for an MWWTP or an IWWTP hinges upon various parameters. These parameters include space limitations, desired effluent quality, frequency of fluctuations in influent flow rate and characteristics [31], and cost considerations. Generally, compared to CAS, the SBR necessitates a smaller footprint and demonstrates greater flexibility in adapting to diverse operating conditions. However, its control can pose challenges compared to CAS, given additional parameters to control, such as the number of cycles within a specified time duration and the optimal duration of each phase. Furthermore, experimental investigations indicated that in treating municipal wastewater, SBRs can exhibit superior 5-day biochemical oxygen demand (${BOD}_{5}$) removal compared to CAS [48]. However, both CAS and SBRs demonstrated the same performance in COD removal [49]. On the other hand, for industrial wastewater, SBRs can result in a higher ${BOD}_{5}$ and COD removal in comparison to CAS [50]. In a theoretical assessment, SBRs demonstrated a near-complete ${BOD}_{5}$ removal for slowly biodegradable wastewater. In contrast, residual ${BOD}_{5}$ content was observed in the CAS effluent. Also, comparable oxygen consumption and sludge generation were observed in both methods. Notably, higher nitrogen removal was ascertained for CAS unless the HRT and the number of cycles per day for SBRs were increased [51]. In terms of cost estimation, a simulation study comparing the implementation of two CAS units in parallel against one CAS unit and one SBR in parallel for a petroleum refinery wastewater treatment plant (PRWWTP) revealed lower expenditures in project construction, operation, energy, and amortization costs for the former scenario [52]. The anticipated costs for materials, chemicals, and maintenance remained consistent between the two configurations. Similarly, in a separate simulation study evaluating the deployment of CAS and SBRs for a WWTP in Tehran, only chemical costs were equivalent for both processes. All other associated costs for SBRs exceeded those for CAS [53]. ASMs and BSMs are applicable to describe the dynamic behaviour of SBRs. Conclusively, the main challenges ahead of dynamic modelling of biological treatment processes, including ASPs and SBRs, are tabulated in Table 2.

### 2.3. Modelling other Biological Wastewater Treatment Processes

While this study primarily concentrates on studying ASPs and SBRs as selected biological wastewater treatment methods, it is important to briefly discuss the modelling of some other biological approaches, previously shown in Figure 1. Among other methodologies, it can be asserted that anaerobic digestion (AD) has achieved a more comprehensive understanding, as evidenced by the development of the standard anaerobic digestion model no. 1 (ADM1) by the IWA [54]. While certain researchers have identified limitations in ADM1 for capturing specific aspects of the AD [55], it remains a valuable tool for effectively describing certain anaerobic processes, such as an anaerobic contact reactor (ACR). Furthermore, some studies have modified the parameters of ADM1 to enhance its applicability to their specific investigated system [56]. Some other studies have employed AI-based methods for modelling AD [57]. Modelling becomes increasingly challenging when addressing processes that encompass both biodegradation and separation processes. Most of the processes listed in Figure 1 involve a synergistic combination of biological and physical processes. For instance, in the case of an aerobic membrane bioreactor (MBR) and an anaerobic membrane bioreactor (AnMBR), the modelling of the process necessitates the comprehensive consideration of various factors and processes occurring simultaneously. This includes consideration of biological metabolism, substance biodegradation, membrane separation mechanisms, the hydrodynamic behaviour of substances, particularly soluble microbial products (SMPs) and extracellular polymeric substances (EPSs) leading to membrane fouling, the specific reactor configuration, operation, and ambient conditions [58,59]. Additionally, it is imperative to consider that these processes interact with each other. For instance, the presence of a membrane influences biomass population and diversity, and membrane fouling can result in short circuits that impact overall membrane performance. Modelling AnMBRs becomes significantly more complex when gas/biogas sparging is introduced as a means of fouling control [59]. In addition, despite numerous efforts to comprehend the behaviour and kinetics of SMPs, certain aspects remain inadequately addressed. On the other hand, the dynamics of EPSs are understudied. It is noteworthy to consider that most studies investigating the behaviour of either SMPs or EPSs rely only on experimental data, which, while valuable, are less effective than examining the actual data [58]. Same as other processes, addressing the difficulties of developing a mechanistic model for MBRs and AnMBRs involves the application of black-box modelling. For example, Li et al. [60] employed three deep learning (DL) methods to forecast the performance of two AnMBR systems in a Japanese WWTP. All DL methods, including machine learning fully connected network (MLFCN), convolutional neural network (CNN), and densely connected convolutional network (DenseNet), demonstrated strong predictive capabilities for AnMBR performance. Notably, DenseNet exhibited the best overall performance. Also, for the modelling of MBRs, ML- and AI-based approaches have been used successfully [61,62]. In another study, Gopi Kiran et al. [63] developed an ANN-based model to predict COD and heavy metal removal in a rotating biological contactor (RBC). It is important to note that the IWA standard models developed for ASMs and ADMs can be incorporated into the mechanistic modelling of processes outlined in Figure 1. For example, a modified ASM1 model was effectively used for the dynamic simulation of a pilot-scale trickling filter bioreactor implemented at the Phu Loc WWTP, Da Nang, Vietnam [64].

### 2.4. Modelling AOP-Based Treatment Processes

AOPs exhibit a complex and nonlinear dynamic for several reasons. The main reason for intricate reaction mechanisms and degradation paths for AOPs is the nonselective reactions of generated ROS with various species extending beyond the target compound. This also involves the nonselective reaction of a ROS with other ROS or even itself, known as the scavenging effect, especially at higher oxidant concentrations than the optimal amount [65,66]. Additionally, the degradation in photo-involved AOPs may occur through both direct and indirect photolysis, adding further complexity to the process dynamic [67,68,69,70,71]. Given the complexity and nonlinearity of AOPs, in studying their dynamics, the tendency is towards utilizing system identification. For instance, Shahwan et al. [72] studied the transient behaviour of the ultraviolet (UV)/$H_{2}O_{2}$ process by modelling the degradation of polyvinyl alcohol (PVA) in a photoreactor. This modelling was conducted by estimating the first-order plus time delay (FOPTD) and the second-order plus time delay (SOPTD) TFs. Both the graphical method and MATLAB system identification toolbox were employed to estimate TFs. In their study, the process response to each step change in the [$H_{2}O_{2}$]_in_/[PVA]_in_ mass ratio, was measured by measuring time-varying pH in the effluent. Subsequently, for each experiment, a separate TF was identified. In most experiments, the FOPTD-TF demonstrated more accuracy than the SOPTD-TF in describing the process dynamic.

Hamad et al. [73] studied the UV/H_2_O_2_ process for PVA degradation as a multi-input multi-output (MIMO) system. In their study, process inputs were inlet feed flow rate (mL/min) and inlet $H_{2}O_{2}$ concentration (mg/L), process outputs were residual $H_{2}O_{2}$ (mg/L) and effluent total organic carbon (TOC) (mg/L), and disturbance was PVA concentration in the feed (mg/L). Experimental data from step change experiments were fitted to a fourth-order state-space model to describe the dynamic behaviour of the process successfully. Recently, Lin et al. [74] used ARX, a nonlinear autoregressive with exogenous input model (NARX) (along with different activation functions), and Hammerstein–Wiener (HW) structures to describe the same system as a single-input single-output (SISO). In their study, the process input was inlet $H_{2}O_{2}$ concentration, and the process output was the effluent pH. Their investigation involved the study of the dynamic system in two scenarios. The first scenario was operating a single photoreactor and the second scenario was the operation of two photoreactors in series. Due to the high nonlinearity of the process, the ARX model showed poor fitness in both studied scenarios (almost 65% fitness). In investigating the single-photoreactor, fitting the experimental data to the HW structure resulted in 68.78% and 69.49% fitness of the training data and validation data, respectively, to the developed model. However, the open-loop stability test and whiteness test failed. In the second scenario [75], fitting the experimental data to the HW structure led to a relatively low fitness level for the training and validation data, with values of 50.6% and 24.76%, respectively. Also, modelling the process using NARX, along with the tree partition function, resulted in the best fit in the single-photoreactor scenario. In that case, the highest fitness of 91.59%, lowest final prediction error (FPE), and lowest mean squared error (MSE) were obtained. However, the best model to describe the process when two identical photoreactors were operating in series was achieved by NARX accompanied by the sigmoid activation function.

The COD removal and colour removal of synthetic textile wastewater in a Fenton process was modelled using the backpropagation function artificial neural network (BPFANN) approach. These models considered the inlet ${Fe}^{2+}$ flow rate, inlet $H_{2}O_{2}$ flow rate, measured pH, and measured oxidation–reduction potential (ORP) in the oxidation reactor as influential factors. Then, to obtain the desired COD/colour removal, ${Fe}^{2+}$ and $H_{2}O_{2}$ dosages were adjusted manually by constantly comparing the predicted process response with the desired one. In fact, the process response was predicted using an ANN model alongside information on monitored pH and ORP of the system and initial dosages of ${Fe}^{2+}$ and $H_{2}O_{2}$ [76]. While that study claimed to address online monitoring and control of the process, it is important to note that the monitoring and computation time intervals were relatively large, allowing the system to reach a steady state. Thus, further research is required to explore the dynamic behaviour of the system during transient states.

Foschi et al. [77] implemented different linear regression approaches, ANN, and two-part ANN (TPANN), to model the UV disinfection process using data from the S. Rocco WWTP, Milan, Italy. The process variables were the concentration of *E. coli* in the influent, the number of operating UV lamps (as a representative of UV intensity), turbidity, and temperature. The process response was the concentration of *E. coli* in the effluent. Djeddou and Loukam [78] modified the performance of the radial basis function neural network (RBFNN) model to predict ozonation disinfection by combining it with the wavelet transformation function. Predicted values for ozone dosing (mg/L) using a hybrid wavelet radial basis function-based neural network (WRBFNN) model showed a good agreement with the actual data. The actual data were obtained from the Oued Al-Athmania drinking water treatment plant. Wang et al. [79] employed the RBFNN to model the nonlinear dynamic behaviour of the primary ozonation step of the water disinfection process in the Xiangcheng drinking water treatment plant (XWTP), Suzhou, China. The RBFNN model was trained using different algorithms, including gradient descent (GD), genetic algorithm (GA), and particle swarm optimization (PSO). Among them all, RBFNN-PSO showed the best convergence and the lowest prediction error. Dongsheng et al. [80] developed the ozonation disinfection model using RBFNN to map the complexity and nonlinearity of the process. Abouzlam et al. [81,82] studied the catalytic ozonation as a single-input multi-output (SIMO) system. In their study, CVs were the concentration of ozone gas in process effluent and the absorbance of UV_340_ at effluent as an indicator of pollutant concentration. The MV was the ozonator inlet power. The nonlinear Wiener model and FOPTD-TF were developed successfully to identify the process. Although employing system identification helped to describe the dynamic behaviour of catalytic ozonation mathematically, the developed model was entirely statistical. Hence, in a later study, Abouzlam et al. [83] investigated the dynamic behaviour of the process using the gray-box approach. In that study, physically meaningful differential equations were developed by applying mass balance equations over the ozonation reactor. Given the existing knowledge of certain time-varying physical parameters derived from experimental data, the Levenberg-Marquardt (LM) algorithm was employed to estimate parameters for the dynamic model. The outcomes of their study demonstrated a good convergence of the LM algorithm. Consequently, a nonlinear mechanistic dynamic model was developed to describe the catalytic ozonation of the synthetic wastewater. In developing mechanistic models for AOPs, a comprehensive understanding of the process is imperative to propose optimal process mechanisms. Also, the identification of the critical reactions responsible for pollutant degradation is essential. This identification often involves estimating reaction rate constants or conducting specific experimental studies. For instance, in AOPs, the implementation of trapping tests is beneficial. In trapping tests, a particular reagent is introduced to the reaction to react with a specific ROS selectively. This helps identify the main ROS responsible for pollutant degradation [20,23,25,84,85,86]. If the contributions of other ROS to the pollutant degradation are negligible, omitting related reactions from the mechanistic model enhances computational efficiency while maintaining accuracy in representing essential reactions. Table 2 outlines the main difficulties in the dynamic modelling of AOP-based wastewater treatment processes and the actions that have been taken to resolve them.

Later, the developed dynamic models for the processes will be utilized in designing appropriate controllers.

## 3. Controlling Treatment Processes

After developing an appropriate dynamic model for the treatment process, the model can be implemented in a process control scheme to regulate the process to reject disturbances or track the trajectory reference. This approach is called process model-based control (PMBC). Table 3 shows the different types of studied and proposed control methods in biological and AOP-based wastewater treatment processes. Table 4 provides details of recent studies on controlling biological and AOP-based wastewater treatment processes.

### 3.1. Controlling Biological Treatment Processes—ASP

With respect to the control of ASP, numerous studies have focused on regulating aeration to control DO, a key factor for maintaining high effluent quality while minimizing energy consumption, which are two main concerns of ASPs. Aeration adjustment is performed manually or automatically using on/off or deviation valves. These valves receive their signals from the controller to minimize the DO deviation from its setpoint [130]. To regulate DO and nitrate concentration in specific points of a biological WWTP, Tejaswini et al. [40] designed and studied the performance of four different types of control approaches, including proportional–integral (PI), fractional order proportional–integral (FPI), MPC, and fuzzy logic control (FLC). In their study, return activated sludge (RAS) and aeration flow rates were manipulated to regulate the process. The performance of designed controllers was evaluated using the BSM1 scheme. Simulation results showed the acceptable performance of the PI controller. However, in the case of FPI, a reduced integral square error (ISE) index, a statistical metric for assessing controller performance, was achieved. Additionally, it was observed that the FLC can significantly decrease ammonia concentration compared to other control methods. The slightest fluctuations in total nitrogen (TN) and ammonia in effluent were also observed for FLC and MPC. The best setpoint tracking was observed for MPC. In fact, MPC can be concluded to be the best control approach in their study because despite the significant influence of FLC on process performance, implementing it can significantly increase the power cost of aeration. In addition, generally, when advanced controllers were introduced, the effluent quality index (EQI) and operational cost index (OCI) decreased significantly by 19.89% and 5.24% for MPC and by 20.9% and 4.63% for the FLC, respectively. Ammonia-based aeration control (ABAC) is a control method in biological treatment. It controls DO in the aeration tank based on measured ammonia in its effluent rather than keeping DO at a constant setpoint [117]. ABAC studies are mainly performed using the BSM1 benchmark and have resulted in decreasing oxygen consumption as well as increasing ammonia removal efficiency [83]. Husin et al. [117] studied and developed an ANN-based ABAC. The BSM1 simulation results demonstrated that, compared to PI-ABAC, ANN-ABAC led to the reduction in the aeration energy cost index (AECI) by up to 23.86%. Additionally, it resulted in an enhancement of the EQI by 1.94% and the reduction in the OCI by 4.61%. Also, satisfactory results were observed in decreasing the number of TN violations in effluent by 28.567% for dry and stormy weather and 40% for rainy weather. Wang et al. [109] developed a control scheme for an MWWTP to maintain the DO and nitrate concentration at their desired setpoints. The iterative adaptive critic (IAC) method was applied in their study to cope with the process complexity. The outcomes revealed a notably superior performance of the developed data-driven IAC control compared to the conventional PID control.

An effective control strategy for simultaneously determining an appropriate setpoint and tracking it is the two-layered hierarchical control (cascade) strategy. First, the developed algorithm in the upper layer determines the lower layer setpoint, and subsequently, the lower layer tracks the setpoint. It has been observed that a multi-loop control strategy significantly increases the performance of WWTP processes [100]. In another study reported by Petre et al. [39], ORP in each of the anoxic and aerobic tanks of the ASP was monitored as a representative indicator of carbonaceous substance content. Their study aimed to achieve a setpoint of 1.5 mg/L for DO in the aerobic basin by adjusting inlet wastewater and air flow rates. Therefore, they designed and simulated an adaptive control to monitor DO and concentration of wastewater in the aeration tank of a multivariable ASP in a real WWTP. Petre et al. [39,87] compared the performance of continuous ABAC with intermittent ABAC for a pilot-scale ASP. In intermittent mode, the setpoint for ${NH}_{4}^{+}$ concentration was defined in the range of 2–5 mg N/L. If the ${NH}_{4}^{+}$ concentration was less than 2 mg N/L, the aeration was turned off through a cascade-PI control loop. Alternatively, if the measured concentration of ammonia was 5 mgN/L or higher, the aeration was kept on. In addition, having a nitrite concentration of zero or less than 3.5 mgN/L was enough to keep the process in air-on mode. During the aeration-on mode, DO was kept at 0.7 mg/L, which is a low-DO setpoint. The aim was to adjust microorganisms to operate in low-DO conditions to save energy. The lower proportional controller adjusted the current DO with a low-DO setpoint by manipulating the flow rate of inlet air. On the other hand, in continuous mode, there was a solo setpoint of 5 mgN/L for ammonium concentration. Also, DO in the inner loop was continuously adjusted between 0.1 and 0.6 mg/L to achieve the ammonium setpoint. Both operational modes exhibited excellent efficiency in ammonia degradation, TN removal, and total phosphorous (TP) removal, surpassing 90%, 60%, and 90%, respectively. However, the intermittent operating ABAC showed better performance in nitrification–denitrification. Another study explored the implementation of hierarchical FLC-PI control by employing BSM1. In that arrangement, the FLC supervised the PI controller. The outcome showed a notable enhancement in DO setpoint tracking. The proposed control improved the quality of ASP effluent by up to 20.3%. In addition, effluent TN and ammonia concentration variations decreased considerably due to the DO setpoint improvement. However, the proposed control scheme showed a minor increase in aeration energy (AE) consumption [88]. A nonlinear model predictive control (NMPC) was formulated in a separate study for DO regulation. This approach involved employing a self-organizing fuzzy neural network (SOFNN) to model the dynamics of the ASP and implementing an adaptive second-order LM algorithm for control of the process. The effectiveness of the proposed control was validated for both varying and fixed set-point scenarios using BSM1 and experimental data [112]. Further, Sadeghassadi et al. [33] proved that employing ANN or fuzzy models as nonlinear predictive models in the MPC structure enhances control performance. In another study, Smida et al. [134] exploited a reduced ASM in the structure of an output feedback predictive control (OFPC) to control nitrogen concentration in the effluent of the ASP. The simulation results demonstrated a good estimation performance. Finally, according to Gu et al. [135], advanced control methodologies, including ANN and fuzzy logic, as well as hybrid approaches such as self-adaptive fuzzy PID control, demonstrated enhanced efficiency in aeration control.

### 3.2. Controlling Biological Treatment Processes—SBR

In SBRs, an optimal control system determines and adjusts the duration of the entire treatment process, the duration of each cycle, and other operating conditions, including DO, based on online measurements of relevant indicators [136]. In the following, a summary of some recent studies on the control of the SBR is presented.

Piotrowski et al. [12] designed a two-layered hierarchical control scheme for an SBR process. A supervisory sequential controller (SSC) controlled the required number and duration of oxidation cycles in the SBR according to a dominant algorithm. In addition, the second algorithm in the SSC, which was an NMPC, controlled the DO level in the SBR tank. The DO level was controlled such that it coped with the fluctuations in the influent flow rate, without altering the effluent quality. In their study, sequential quadratic programming (SQP) was implemented to solve the NMPC optimization problem. Their study was an attempt to improve the performance of the current SBR control system in a WWTP in Poland. The results of their study showed that the performance of the proposed control system was promising. In another study by Dries [31], aeration in an SBR was controlled through an on/off method by monitoring DO in the system over time and maintaining it at 2–3 mg/L. Also, the oxygen uptake rate (OUR) was determined online by calculating the slope of the DO curve. After a minimum of 30 minutes of aeration, the duration of the aerobic stage was determined based on the difference between the current OUR value and its value at the past sampling time. Achieving OUR of 15 mg/L.h and a maximum OUR difference of 1 mg/L.h between two sequential sampling times were determined as the aeration ending point indicator. Monitoring ORP during the anoxic filling of an SBR revealed crucial information. The point at which the slope of the ORP curve versus time changed by −50% signified the termination of the anoxic denitrification stage. This transition indicated the starting time for the aeration, which was the subsequent phase. It should be considered that at the initial point of the anoxic filling phase, DO was less than 0.5 mg O_2_/L, and controlling the SBR through monitoring the reduction in the ORP slope started right after filling the tank with a minimum amount of wastewater and continued to the ending point of the anoxic phase. In addition to the ORP reduction trend, reaching the maximum capacity of the SBR tank was another indicator for terminating the anoxic filling stage [31]. In a study by Dries [31], the Nessler, cadmium reduction, and gravimetrically methods were used to measure ammonium, nitrate, and MLSS, respectively. The results of their study demonstrated that implementing the proposed SBR control system increased the efficiency of the process and saved time and energy. The process was modified by adjusting operation conditions, including the ratio of the fed wastewater to microorganisms, duration of SBR phases, and rate of exchanging the volume. The adjustment was performed based on the characteristics of activated sludge (AS). The results of that study have encouraged industries to implement SBRs for treating high-ammonia wastewater. Furthermore, in controlling the nitrification process in a lab-scale bioreactor, van Rooyen et al. [137] kept the biological reaction rate at its maximum amount by monitoring and controlling the pH of the process. The pH-based control system was used because of the importance of pH in nitrification. Indeed, on converting each mole of ammonia to nitrate, one mole of H^+^ was produced. Consequently, it was concluded that pH is a reasonable indicator of process activity. To control the pH in the system such that the process operated at its highest yield, whenever a pH change was observed, hydroxide (through dosing potassium hydroxide (KOH)) was added to the system to compensate for the pH drop. After a while, observing a constant pH indicated ammonia extinction in the bioreactor and marked the proper time to empty the tank and refill it with a new substrate. Hydroxide dosing in their study was controlled by an FB-PI-control scheme after each pH reading at 30-min intervals.

### 3.3. Controlling other Biological Treatment Processes

Although exploring control of other biological treatment processes than ASPs and SBRs is not the focus of this study, a brief mention of such processes is included in this section and Table 4. The primary control objectives for most of the biological wastewater treatment processes outlined in Figure 1 are to maintain the effluent quality and/or minimize costs, energy consumption, and greenhouse gas (GHG) emissions. Effluent quality control in biological filter or membrane-based processes, such as trickling filters, RBC, MBRs, or AnMBRs, is more challenging compared to processes that only involve biological treatment. The optimal control of these processes requires controlling both the filtration and biological processes. Adjusting process parameters such as HRT, sludge retention time (SRT), carbon-to-nitrogen ratio, alkalinity, pH, or temperature can regulate the biological process [138,139]. On the other hand, fouling control can be achieved in an open-loop or closed-loop manner by manipulating flux, initial time and duration of backwashing, relaxation, and permeation stages or scouring gas/biogas purging [59,138,139]. Mahmod et al. [62] explored several ANN-based structures to identify the most effective one in capturing the dynamic behaviour of a pilot-scale MBR system. The results of their investigation revealed that RBFNN exhibited remarkable accuracy in predicting the process output. Subsequently, the developed RBFNN model was integrated into an internal model control (IMC) structure to regulate flux and transmembrane pressure (TMP) by adjusting the voltage of the permeate pump. The implemented RBFNN-IMC system demonstrated efficient disturbance rejection and precise tracking of the setpoint trajectory. In another study, Robles et al. [105] designed a multi-loop control system for an AnMBR by incorporating both on/off and PID controllers. Throughout their investigation, each process parameter was controlled by manipulating an appropriate input parameter in an FB-SISO loop. This led to the formation of a closed multi-loop system capable of effectively regulating the process. The performance of a laboratory-scale up-flow anaerobic fixed bed reactor was controlled through a cascade of P controllers supervised by a rule-based controller. The pH, gas flow rate, and methane content in the effluent were the CVs, while the MV was the inlet wastewater flow rate [102]. Mannina et al. [103,132] designed a cascade of PI controllers to regulate ammonia and nitrite concentration by manipulating aeration in a series of AD and MBR bioreactors. In another study, García-Diéguez et al. [104] designed a cascade of PID controllers to control volatile fatty acid (VFA) concentration and methane flow rate at the outlet of a pilot-scale up-flow sludge bed filter by manipulating the inlet feed flow rate. The simulation results demonstrated the effectiveness of the proposed control, showcasing its capability to successfully reject even severe disturbances. In conclusion, beyond addressing effluent quality, the efficiency of biogas generation, and the control of fouling are essential considerations in the control of AD-based and filtration-based biological treatment methods, respectively. Also, Klaus et al. [123] implemented a pH-based control for the aeration of a deammonification moving bed biofilm reactor (MBBR) to enhance the process performance. Indeed, by monitoring effluent ${NH}_{4}^{+}$ concentration, conductivity, and pH, it was observed that the pH is the best representative of the residual alkalinity, indicating the activity of nitrifier bacteria. Conclusively, implementing pH-based aeration control for deammonification MBBR prevented over-aeration and under-aeration. This improved ammonia removal by up to 90%. Also, alkalinity depletion was effectively prevented.

### 3.4. Controlling AOP-Based Treatment Processes

The main challenges associated with implementing AOP-based processes in full-scale include their high operating costs and the risk of effluent quality violation in the presence of disturbances, which is attributable to their short time delay. As a result, reliable control systems must be designed and implemented for these processes to maintain effluent quality and reduce operating costs. Some studies regarding this matter are summarized in the following paragraphs.

Hamad et al. [73] designed an MPC scheme to regulate the PVA degradation process in two UV/H_2_O_2_ photoreactors in series, by integrating the state-space model of the process. The results of their study demonstrated the effective performance of the proposed control in achieving setpoint tracking for effluent TOC and residual $H_{2}O_{2}$. This was accomplished through the regulation of inlet wastewater flow rate and inlet $H_{2}O_{2}$ concentration. Lin et al. [75] developed dynamic models using system identification techniques to map the nonlinearity of the UV/H_2_O_2_ process. Later, they employed the best-developed models in designing controllers. Different controllers (P, PI, and PID) were tuned based on developed ARX and NARX-sigmoid models for two photoreactors in series. It was observed that ARX-PID and ARX-sigmoid-PID have the best performance of all. However, the response of the NARX-sigmoid-PID was sluggish, and the ARX-PID had a considerable overshoot [36,37,75].

One crucial matter in water disinfection using ozonation is adjusting ozone dosing. As a result of optimal ozone dosing, the disinfection performance is maintained at a high level, and the probability of producing bromate by-products at high residual ozone concentrations stays low. Additionally, this adjustment is cost-effective as it prevents ozone wastage and conserves both oxygen and electricity. Wang et al. [79] designed an adaptive NMPC control for the main ozonation step of the water disinfection process in an XWTP, Suzhou, China. They utilized a developed RBFNN model in the structure of NMPC. The control goal in their study was to maintain the residual ozone in the effluent (mg/L) and ozone exposure (mg/L min) at their desired values by adjusting the inlet ozone gas flow rate (L/min) and the concentration of ozone in the inlet gas (mg/L). In the designed adaptive control, whenever the root mean square error (RMSE) was greater than 0.2, weights of the RBFNN model were updated using the recursive least square (RLS) algorithm. The performance of the adaptive RBFNN-MPC was compared with the performance of a PI control scheme. Based on the MATLAB simulation results, the MPC controller exhibited a smaller overshoot, shorter settling time, and reduced integral of absolute error (IAE) compared to the PI controller. Also, they observed when the adaptive RBFNN model was embedded in MPC control, closed-loop performance was much better than embedding the fixed RBFNN in the MPC structure. Experimental data obtained after the implementation of the proposed control strategy in XWTP verified the excellent performance of adaptive RBFNN-MPC. In another study, Dongsheng et al. [96] deployed the same developed RBFNN model, describing the ozonation disinfection, in an IMC scheme to control the constant contact time of ozone with the water by adjusting the ozone dosage. Based on their study, determining a constant setpoint for ozone exposure is a more efficient control strategy than defining a constant setpoint for ozone dosing or for the concentration of residual ozone in the effluent. In another study, Dongsheng et al. [80] implemented an MPC control based on a process model developed using the support vector machine (SVM) method to describe the same ozonation disinfection process. In their study, the ozone dosage was manipulated to maintain the contact time constant, in the presence of fluctuations in influent water characteristics. Both IMC and MPC control outperformed PI control in managing the ozonation disinfection process. The addition of a catalyst to the ozonation process enhances its performance by increasing the efficiency of both direct (reaction of organics with ozone) and indirect (reaction of organics with generated ROS) ozonation reactions. Abouzlam et al. [81,82] implemented catalytic ozonation to remove paranitrophenol from synthetic wastewater. The main problem in advance of using catalytic ozonation for wastewater treatment is its high operating cost due to the substantial electrical and oxygen consumption in the ozonator. To minimize the operating cost and maximize the removal efficiency, the amount of generated ozone can be optimized by manipulating the ozonator inlet power. Thus, Abouzlam et al. [81,82] proposed an FB control scheme. The optimal gain values of controllers were calculated using the developed TFs along with the linear quadratic (LQ) algorithm. The proposed optimal control showed a good performance in regulating the process when positive or negative step changes were applied to the inlet pollutant concentration.

Lin et al. [133] proposed a manual control scheme to monitor and control either UV or UV/TiO_2_ processes in a lab-scale photoreactor to disinfect actual wastewater samples obtained from the Miao-Li City sewer system in Taiwan. The MV and CV were the inlet wastewater flow rate (ml/min) and the total coliform counts (CFU/100 ml) in the effluent, respectively. The initial wastewater flow rate was estimated using the developed relationship between UV dose (μW/cm^2^) and the desired decrease in total coliform count (CFU/100 ml). By knowing the total coliform counts in influent (CFU/100 ml), contact time (s), and by online monitoring of pH, ORP (mV), turbidity (NTU), temperature (℃), UV intensity (W/m^2^), and colour (ADMI) in the photoreactor, coliform counts in the effluent (CFU/100 ml) were predicted. The prediction model was developed using the BPFANN method. The error was generated by comparing values of predicted coliform counts in the effluent and its setpoint. If the error was negligible, the process was kept operating at the current inlet flow rate. If the error was considerable, the inlet flow rate was adjusted manually to push the CV toward its setpoint. By developing and utilizing the BPFANN model in the control scheme, the energy demand decreased by 13.2–15.7 percent.

Despite the acknowledged advantages of all studies in the control domain, it is noteworthy that a substantial portion of them, except some [79,87,99,101,102,107,108,129,131,133], are confined to simulation stages, lacking real-case implementation and verification. Consequently, prevalent, applied control strategies in WWTPs primarily involve PID and MPC, with the occasional integration of fuzzy logic, ANN, and adaptive control in specific instances.

Table 5 summarizes the motivations and limitations of developing process control for biological and AOP-based treatment methods. It must be considered that achieving reliable control requires accessing real-time process data. This matter is discussed in the next section.

## 4. Monitoring Treatment Processes

Investigating the online measurability of process variables is crucial when designing a reliable control system for a biological or AOP-based wastewater treatment unit. Real-time data are essential for understanding the process status and enabling the control system to promptly regulate processes as needed. When process variables are not measurable online, surrogate variables or other correlated online measurable parameters could be utilized for parameter estimation. Accordingly, measuring and monitoring process parameters can be performed using hardware or software sensors. Apart from its role in process control, data acquired from hardware or soft sensors are essential for calibrating and validating ASMs and ADMs tailored to each specific application. In the following, hardware and soft sensors will be discussed in detail.

### 4.1. Hardware Sensors

Hardware sensors are devices that indicate a characteristic of a medium and report it instantly via an understandable analog or digital output. Based on their operation, generally, three classes of sensors are commonly used in WWTPs, including optical sensors, biosensors, and ion-selective electrodes (ISEs) [13]. Employed sensors in the control system should be highly reliable with the slightest noise, deviation, and drift [40]. The simplest and most highly implemented hardware sensors in MWWTPs are thermometers, pressure gauges, liquid level sensors, flowmeters, pH meters, electrical conductivity meters (EC), ORP meters, and total suspended solids (TSS) probes to monitor the general properties of the fluid and settleometers to monitor the settling properties of the sludge [13,140]. TSS sensors are mostly used as an alternative method for measuring MLSS, replacing lengthy laboratory analysis [13]. Additionally, the most common sensors/analyzers to monitor the biological treatment processes are DO meters, UV spectrophotometers, fluorescence, online COD meters, TOC analyzers, TN analyzers, and ISE sensors for measuring ${NH}_{4}^{+}$, ${NO}_{3}^{-}$, and ${NO}_{2}^{-}$ concentrations [13,140]. Also, short-term biochemical oxygen demand (BOD_st_) can be measured by implementing biosensors or online respirometers, such as RODTOX [140].

The innovative sensor-driven control strategies have improved nutrient removal in WWTPs by 10% and resulted in energy savings [13]. A survey of 90 small and medium enterprises (SMEs) operating their own WWTPs in Flanders, Belgium, showed that the most employed sensors in IWWTPs were the DO meter and the pH meter, available in 96% and 69% of locations, respectively. Other sensors were rarely observed in surveyed IWWTPs [141].

Depending on the location of the sensors, real-time data monitoring provides valuable information about the influent characteristics, operating conditions in the reactor, or effluent characteristics. This information can be interpreted and used in FF or FB control schemes to control the wastewater treatment process. As a result, the effluent quality limits are met, and the material, time, and energy consumption are decreased effectively. Also, online monitoring offers a notable reduction in labour costs by obviating the necessity for collecting and processing samples and associated manual work inherent in offline analyzer tests. Additionally, it eliminates the need for dedicated offline analytical laboratories [142].

In aerobic biological treatment processes, the stage of biological reactions can be determined by monitoring the concentration of nitrate, nitrite, or ammonium, using sensors. The termination of the denitrification stage can be determined by finding the ‘nitrate knee’ through online monitoring of ORP or indicating the ‘ammonia valley’ by online monitoring of pH. The ammonia valley is observed when the pH slope changes. Then, this obtained information, along with DO monitoring, can be used in an FB control loop to regulate the aeration rate [37]. The ${NH}_{4}^{+}$ sensor has been especially used in ABAC to adjust the aeration of ASP through monitoring and controlling the ${NH}_{4}^{+}$ concentration [87]. Inventing and implementing the nitrate, nitrite, and ammonium sensors in the biological treatment stage to regulate aeration is considered the most remarkable achievement in recent years regarding improving the quality and consistency of ASP effluent [13].

Also, the online respirometry data can be implemented for the purpose of aeration or RAS rate regulation in the biological treatment process [12,143]. Respirometry rate represents the digesting bacteria OUR due to their metabolic processes. OUR can be monitored using online respirometer probes [143]. However, to have a more effective control, knowing the MLSS value of the AS and employing it to determine the specific oxygen uptake rate (SOUR) is required. Also, by utilizing the data obtained from inline respirometry, various parameters can be assessed based on the specific WWTP. These parameters include the permissible maximum and minimum DO levels within the aeration tank. Moreover, the optimal utilization of tanks can be ascertained using the same data set. The other information from respirometry is understanding the required time to reach the endogenous respiration stage. Then, this information is used as an index to determine and control the required HRT in the aeration tank [143]. One advantage of measuring respirometry in the ASP is its shorter analyzing time, which makes it more reliable compared to COD and ${BOD}_{5}$ tests. Also, respirometry represents the process much better by providing information on both nutrient removal and microorganism growth [144]. Respirometry tests can be performed in a batch system or a continuous liquid flow. In online respirometry, AS is sampled at 1.5–3 h intervals.

Due to the presence of a variety of compounds in wastewater, surrogate parameters are usually measured to represent the strength of influent wastewater or the quality of the effluent. These parameters include ultraviolet–visible (UV-VIS) spectroscopy, fluorescence spectroscopy, TOC, COD, ${BOD}_{5}$, TN, and TP. However, measuring the parameters through conventional sensors/analyzing methods is both time and chemical demanding. Thus, online TOC, COD, ${BOD}_{5}$, and TP sensors have been developed, and efforts are being directed toward their refinement to enhance their accuracy and cost-effectiveness [13]. Some challenges lie ahead for commonly used sensors in WWTPs include the limited measuring range, restricted lifetime, the need for regular recalibration, and the possible interference of other parameters with the measured value. Addressing these issues is crucial for the effective functioning of wastewater treatment processes [145]. Consequently, many continuing studies aim to address the problems regarding hardware sensors.

For instance, Duan et al. [146] have developed a thin-film electrochemical sensor by incorporating a copper nanoparticle (Cu-NP)-modified carbon-silica (C/${SiO}_{2}$) for online COD measurement of the MWWTPs influent. The acceptable performance of the proposed electrochemical sensor was approved by comparing measured values using the sensor with COD readings from the dichromate standard method. They claimed the proposed COD sensor works accurately with low maintenance.

Despite the dramatic evolution of hardware sensors in terms of precision and endurance, the response time of some of them must be reduced to make them suitable for real-time monitoring and control [145]. The short response time for a sensor is crucial, particularly when monitoring and controlling AOP-based treatment processes. This is because the inherent time delay of these processes is relatively short. For good reasons, highly reliable, expensive sensors demanding high maintenance costs might be used in a WWTP for calibration, optimization, or periodic audits. However, as a cost-effective approach, real-time monitoring of the WWTPs process parameters for control purposes is performed by implementing other inexpensive, simple, and reliable sensors [140]. For instance, the high cost of available online UV-based nitrate and online ammonium sensors justifies the ongoing use of affordable sensors such as pH, DO, and ORP for controlling biological treatment units [31].

Some parameters discussed for the monitoring of aerobic processes, such as pH and ammonia concentration, are also applicable for AD-based processes. However, monitoring and controlling other parameters such as the quantity and composition of generated biogas, and the concentration of specific products, such as VFAs, are crucial too. Thus, various sensors, biosensors, and analyzers based on titration, chromatography, spectroscopy, and electrochemistry methods have been proposed in various studies to address these monitoring requirements [147]. Moreover, for fouling control in membrane-based biological processes, monitoring TMP through pressure gauges and pressure transducers is advantageous.

Another recent development in real-time monitoring pertains to transmitting measured data through wireless sensor networks (WSNs) facilitated by the Internet of Things (IoT). This technological innovation provides operators at diverse remote stations with data that closely approximate real-time information [148]. This technology has undergone examination in a developmental phase nearing market readiness. Upon its introduction to the market, it is poised to augment the accessibility and convenience of real-time data.

In large WWTPs with large bioreactors and AOP reactors, another parameter that influences the control system performance is the location of sensors. Sensors should be located at suitable points so that the measuring is sufficient to represent the target parameter. For instance, placing the respirometer at the inlet of the aeration tank represents the strength of the inlet wastewater. However, placing it at the inlet of RAS represents the viability of the returned sludge to the tank [143]. In addition, fouling-sensitive sensors, such as DO meters, should be installed in locations with the lowest fouling risk [140]. In addition, sensor redundancy, wherever it is possible, can decrease the risk of mismeasurement resulting from the malfunction or failure of inexpensive sensors such as pH meters [140]. Lastly, regular maintenance and cleaning of sensors are required to obtain reliable data.

### 4.2. Soft Sensors

Sometimes, the rapid measurement of some process variables is limited to the lack of online hardware sensors, the long measuring response time, noisy measurement, high maintenance demand, or the high purchasing price of available hardware sensors [41]. Soft sensors are implemented to overcome these limitations by indicating process variable value through measuring state variable(s) using the cost-effective sensor(s). Essentially, the value of a process property that cannot be directly measured is inferred by substituting the output of one or several sensors into a mathematical estimation expression. This expression is derived from various modelling approaches, including mechanistic, system identification, or various AI-based DDM methods, describing the correlation of the desired variable with other measurable parameters [13,145,149].

In the field of wastewater treatment, measuring the concentrations of pollutants to evaluate the rate of abatement or to predict the required operating conditions to achieve the desired removal is crucial. However, measuring the concentrations of most compounds is carried out only offline. In such cases, the indirect estimation of organic concentrations through the online measurable surrogate parameters is beneficial and necessary. The main surrogate parameters are UV-VIS spectroscopy, fluorescence spectroscopy, and other surrogates such as TOC, COD, and nitrate formation [150]. The last three are not online measurable, or if so, their operating range is limited while their cost and maintenance are significant. Thus, a good solution is to estimate the value of the parameters by using a well-developed mathematical model describing their relationship with those correlated parameters that are online measurable with cheap sensors such as pH, DO, and ORP [136].

Estimating certain parameters through validated models could also decrease the capital and maintenance costs of a WWTP by minimizing the number of implemented hardware sensors. Even the relationship between surrogate parameter values at the process inlet and their corresponding values at the outlet could be described by developing appropriate mathematical expressions. In such circumstances, the process of measuring surrogate parameters at specific points can be avoided to reduce expenses and minimize labour. For instance, predictive models for effluent COD, ${BOD}_{5}$, and TSS of the biological treatment of the Doha West WWTP were developed using the ANN approach and based on measured surrogate parameters in the plant influent [151]. Based on calculated MSE and R^2^ values for each developed model, the best estimation for TSS_eff_, BOD_5,eff_, and COD_eff_ were achieved when they were modelled solely as dependents on COD_in_. Estimating COD_eff_ as a function of TSS_in_, BOD_5,in_, and COD_in_ parameters also resulted in a high R^2^ and low MSE. Nevertheless, the prediction performance of the model did not significantly surpass the model that correlated COD_eff_ solely with COD_in_. Conclusively, TSS_eff_, ${BOD}_{5,eff}$, and COD_eff_ can be accurately estimated only by monitoring COD_in_ [151]. Some studies developed models to estimate some process parameters in WWTPs based on other measured parameters data are shown in Table 6.

Fluorescence and UV-VIS spectroscopy have been used in AOPs, particularly the ozonation reaction, as real-time surrogate parameters to indicate the removal rate of trace organic contaminants (TrOCs) from wastewater [150,157]. In wastewater, most dissolved organic matters (DOMs) having multiple conjugated double bonds, particularly hydrophobic and aromatic matters having a C=C and a C=O bond, absorb UV light well at the wavelength of 254 nm. The UV absorbance ratio for these compounds is proportional to the concentration of organic compounds present in the medium. Therefore, the percentage of organic removal in some AOP-based treatment reactions can be determined by comparing the UV_254_ absorbance at reactor effluent with its amount at reactor influent. Depending on the organic compounds, process, and operating conditions, $∆{UVA}_{254}$ correlates to the amount of organic removal through a linear [158,159,160,161,162,163,164,165,166,167], S-shaped [168,169,170,171], logarithmic [172], exponential [172], linear biphasic [159,161,173], or other mathematic relation that need to be developed experimentally. In addition, the optimal UV absorbance for some organic compounds occurs at wavelengths other than 254 nm. This optimal wavelength can be determined by referring to the open literature or by conducting preliminary experiments to measure UV absorbance by the target component at different wavelengths to find the wavelength at which the highest absorbance happens. For instance, Wert et al. [165] and Miklos et al. [174] quantified the colour in their samples by measuring UV absorbance at 455 nm and 436 nm, respectively. The treatment process in the conducted study by Wert et al. [165] was ozonation, and in the conducted study by Miklos et al. [174], were UV/H_2_O_2_, UV/PDS (peroxydisulfate), and UV/chlorine.

Si et al. [158] measured the UV absorbance of samples obtained from the effluent of combined ozonation and ultrafiltration treatment reactors. These measurements were conducted at various wavelengths, including 254, 258, 260, and 280 nm. The purpose was to estimate the concentrations of substances with conjugated double bonds, aromatic unsaturated organics, nucleic acids, and aromatics, respectively. Li et al. [169,175] measured the UV absorbance of their samples at 280 nm and 366 nm to estimate the concentrations of organics.

Furthermore, some researchers believe UV spectroscopy at a fixed light wavelength does not give sufficient information about the reaction and converted chemicals compared to UV spectroscopy at an interval of light wavelengths. To this end, differential absorbance spectra (DAS) and absorbance slope index (ASI) were introduced [168,176]. These two concepts are beneficial for estimating compound concentrations. Estimating compound concentrations is achievable by substituting values of measured UV absorbance at different wavelengths in verified empirical equations. Audenaert et al. [177] implemented the ASI approach to estimate the molecular weight of natural organic matter (NOM) in the effluent of ozonation and UV/H_2_O_2_ processes. UV-VIS spectroscopy was also implemented in a study by Qin et al. [178]. They developed a relationship between each COD, TSS, and oil and grease (O&G) concentration with UV-VIS absorbance and turbidity of the inlet and outlet of an electrocoagulation–electroflotation unit. The method used in modelling was boosting-iterative predictor weighting-partial least squares (Boosting-IPW-PLS). Their results demonstrated that the developed models are reliable. Consequently, they highlighted the potential of using inline UV-VIS spectrophotometers and turbidity sensors alongside the verified empirical estimation models to accurately estimate concentrations of COD, TSS, and O&G in real time.

In addition to UV-VIS spectroscopy, to analyze a sample with low concentrations of organic compounds, especially organic dyes and compounds containing aromatic groups, fluorescence spectroscopy is applicable. The fluorescence even provides more accurate information than UV-VIS spectroscopy in the case of substance identification. Estimating the organic removal in some cases is achievable by evaluating the changes in integrated volume under the excitation–emission matrix (EEM) through comparing the fluorescence spectroscopy results of wastewater influent with the results of effluent [150]. This value is called the difference of total fluorescence (∆TF). Studies on the TrOC removal by ozonation show a linear [160,162], a linear biphasic [173], a logarithmic [172], and an exponential [172] correlation of ∆TF with the concentration of TrOC in the effluent. In addition to ∆TF, other fluorescence spectroscopy-based methods, including fluorescence index (FI), peak A, peak B, peak C, peak T, and parallel factor analysis (PARAFAC), are used to estimate the fluorescent compound concentration in a matrix. These later methods have been used in some ozonation [170,175,179] and UV/H_2_O_2_ [180] studies to measure the concentration of specific organics in the reactor influent and effluent. Considering the main substances that are removed in each step of a WWTP, during biological treatment and tertiary treatment, the peak T and peak C fluorescence, respectively, decreased [181]. It has even been observed that by knowing the information on the peak T fluorescence of the municipal wastewater sample through developing proper statistical equations, ${BOD}_{5}$ content can be estimated [181].

Some studies have used a combination of UV-VIS and fluorescence spectroscopy to determine the target parameter. For instance, Gerrity et al. [163] developed a correlation between UV_254_/fluorescence absorbance and the pathogen concentration in the effluent of an ozone disinfection unit. As a result, monitoring the efficiency of the process and quality of the effluent was possible through the online measurement of UV_254_/fluorescence absorbance as representers of pollutant and pathogen concentrations. Depending on the target pollutant, the same approach can be developed and calibrated to monitor the effluent quality of other AOP-based treatment processes. Also, for fouling control in membrane-based biological treatment methods, considering that the primary cause of fouling is the accumulation of SMPs and EPSs, it is beneficial to monitor and control their concentration using established offline methods or through UV/fluorescence spectroscopy techniques [182]. The developed soft sensors can subsequently be deployed in the supervisory control and data acquisition system (SCADA) of the WWTPs [149].

Finally, the response time of a soft sensor is a summation of the hardware sensor response time and the model computation time. Hence, having a fast response time for a hardware sensor becomes even more crucial when hardware sensors provide data for a soft sensor model [145].

## 5. Conclusions

This review highlights the importance of designing control strategies for wastewater treatment systems, focusing on selected biological and AOP-based methods, to improve their operations. The aim is to continuously maintain desired effluent quality to meet environmental regulations and minimize operating costs. In the second part, this study emphasized the understanding of the dynamic behaviour of the processes, the first and crucial step in designing and developing an effective control scheme. Challenges ahead of dynamic modelling of biological and AOP-based wastewater treatment processes were discussed. It was shown that dynamic models can be developed based on the mechanistic aspects and the physicochemical knowledge of the process, known as mechanistic models, or completely based on experimental data through system identification. It was discussed that due to the longstanding and well-established nature of biological treatment methods, IWA has developed standard models, including ASM1, ASM2, ASM2d, ASM3, ASM4, ASM7, and ADM1, to describe the dynamical behaviour of aerobic and anaerobic biological processes. Some processes, such as ASPs and SBRs, can be fully described based on these standard models. However, to describe filtration-based biological processes, including AnMBRs and MBRs, a more comprehensive model is required to cover both the biological and physical aspects of the process. In addition, it was highlighted that standard models must be calibrated for each WWTP using real data obtained from that specific WWTP. Furthermore, it was outlined that for both biological and AOP-based treatment methods, DDMs based on black-box system identification or AI-based models can result in precise predictive models, particularly in instances where process dynamics exhibit significant nonlinearity. Even though the black-box models may not describe the process mechanistic behaviour in detail, they can accurately anticipate the response of a system to unexpected disturbances. Finally, selecting calibrated mechanistic models or DDMs must be conducted according to the system complexity and control goal. It involves a trade-off between having a comprehensive process description, which requires extensive knowledge, calibration, and verification, or using DDMs that rely on substantial data but may lack specific meaning as they are primarily mathematical representations of the process. Also, it must be considered that either mechanistic models or DDMs are valid for a limited operating range, corresponding to the defined constraints during the model development phase.

Part three reviewed the most common process control strategies in WWTPs. Also, the most recent studies and their findings on controlling biological, and AOP-based wastewater treatment processes through different control strategies were discussed in detail. It was debated that while numerous studies have demonstrated promising outcomes in controlling treatment processes, their applicability remains primarily confined to the simulation stage. This fact poses challenges in translating these achievements to full-scale implementation. A notable impediment arises in the necessity to validate the performance of proposed control strategies through the practical deployment of hardware controllers. Additionally, their inherent complexity and associated cost hinder the widespread adoption of advanced control strategies, prompting many WWTPs to adhere to conventional PID controllers. Aside from financial constraints, the proper selection of a control scheme for the validation and implementation in a wastewater treatment process necessitates a deliberate consideration of the desired control objectives. Contingent upon the specific control goals, the optimal control strategy may diverge, ranging from linear conventional controllers to intricate advanced, AI-based, hybrid, or hierarchical configurations. For instance, to have a control scheme with high performance to ensure the least deviation in effluent quality for ASPs, SBRs, MBRs, AnMBRs, or AD, a hierarchical (cascade) control scheme is recommended. This approach entails monitoring and controlling additional parameters, such as ammonia concentration, in conjunction with traditional DO. It utilizes advanced control methods as supervisory controllers such as FLC, MPC, NMPC, rule-based or ANN to determine the setpoint of the lower control loops. The lower controllers typically are simpler ones such as on/off, P, PI or PID. Also, implementing an adaptive model with varying parameters in the structure of the controller will yield better control performance while the dynamic of the process is varying. Similarly, the choice of the control scheme for AOP-based treatment processes is upon the control goal, ranging from linear approximations such as ARX-PID to more sophisticated methods such as NMPC. Also, considering the short time delay of AOP-based treatment processes and process uncertainties, a cascade of FF and FB controls was discussed as a sufficient control strategy. Generally, by analyzing the results of other studies, it was concluded that multi-loop hierarchical (cascade) control is the optimum control design for most wastewater treatment processes and enhances their performance. In addition, the limitations of controlling treatment processes, including insufficiently developed models, the presence of interactive processes, limitations in control final elements, and limitations in the availability of real-time data and sensors, were discussed.

The necessity of the availability of real-time data to achieve reliable control was discussed in part four. It was observed that this can be reached by either implementing proper and accurate hardware sensors in suitable locations of the process or developing and implementing soft sensors. Also, surrogate parameters such as TOC, COD, ${BOD}_{5}$, TN, TP, UV-VIS absorbance, and fluorescence absorbance can be considered as representatives of substances concentration. Depending on the process and the characteristics of the target wastewater to be treated, selecting the monitoring parameters must be performed wisely. For instance, although photo spectroscopy provides real-time data, if the medium is highly concentrated with variant temperature, the measured data might be subjected to a considerable error, and sensor fouling will be a further problem. Addressing this issue is possible by redirecting a small part of the effluent to a separate pipeline and mixing it with clean water using an appropriate dilution factor before conducting spectroscopy. Also, the mismeasurements caused by temperature violations can be resolved. This involves placing thermometers at appropriate locations and adjusting the measurements to account for the impact of the real-time temperature. Also, in the case of UV-spectroscopy, finding the best wavelength, especially when wastewater contains different pollutants, is a challenge. The limited operating range or long response time of some new sensors are also some challenges ahead of hardware sensor implementation in the process control system. On the other hand, although soft sensors provide users with well-established mathematical models, estimating the desired parameter, it must be considered that the response time of a soft sensor is the summation of hardware sensor response time and computation time. As a result, these two response times must be minimized. Another significant finding underscores that despite the recent advancements in hardware sensor technology, further endeavours are required to enhance their cost-effectiveness. This improvement is pivotal for facilitating the widespread transition of WWTPs from conventional sensors to these state-of-the-art alternatives. The last noteworthy discovery in real-time monitoring pertains to the prospective integration of WSN and IoT as a future mechanism for transmitting and controlling measured data through sensors.

To achieve a robust and reliable control in WWTPs, especially regarding biological and AOP-based treatment approaches, it is recommended that another study be conducted on available actuators, final elements, and the criteria for choosing the most appropriate one. Also, planning proper maintenance and cleaning schedules for hardware sensors should be considered. In the fourth section of this study, the potential surrogate parameters for process monitoring in WWTPs were discussed. However, it is crucial to acquire a thorough understanding of the latest technological advancements associated with analytical techniques and devices for measuring each surrogate parameter. Such information is beneficial in empowering decision-makers to select technologies that precisely align with their needs. Consequently, conducting a comprehensive and up-to-date exploration of the state of the art of surrogate parameter technologies is recommended. In addition, further research is recommended to delve into the potential unique challenges associated with the real-time monitoring of diverse organic and inorganic pollutants. Exploring the classification and specific characteristics of each pollutant class and type can be beneficial for addressing the distinct challenges posed in dynamic modelling, monitoring, and control processes. Finally, although this manuscript, in addition to focusing on ASPs and SBRs as selected biological wastewater treatment for this study, attempted to briefly discuss some other biological methods, it is essential to acknowledge the wide range of biological methods. Therefore, it is recommended to conduct a focused review on modelling, control, and monitoring each of these processes in MWWTPs and IWWTPs.

## Figures and Tables

**Table 1:** The main ASMs in wastewater treatment developed by the IWA.

Model	Description	Application
ASM1	Biological conversion of organic matter into biomass and carbon dioxide in ASPs	Most widely used model to design/simulate conventional ASPs
ASM2	Extension of ASM1, includes the conversion of nitrogen and phosphorus compounds	Predicting behaviour of nitrogen removal processes
ASM2d	Extension of ASM2, includes additional details and factors affecting the performance of the treatment process	Predicting process performance and behaviour of nitrogen removal
ASM3	Extension of ASM2, includes phosphorus removal through biological processes	Predicting behaviour of nitrogen and phosphorus removal processes
ASM4	Extension of ASM2, includes phosphorus removal through chemical precipitation	Predicting behaviour of nitrogen and phosphorus removal processes
ASM7	Comprehensive model, combination of ASM1, ASM2, ASM4	Predicting behaviour of nitrogen and phosphorus removal processes

**Table 2:** Challenges ahead of dynamic modelling of biological and AOP-based wastewater treatment processes.

Wastewater Treatment Process	Challenges Ahead of Dynamic Modelling	Actions Taken
Biological	Complex and interactive combination of biodegradation and physical processes; Complex behaviour of microorganisms in presence of process disturbances; Non-linearity of the process; Large time delay; Calibration requirement of ASMs and ADMs for each specific treatment unit; Need for tailored calibration methods with specific data sets for each type of ASMs and ADMs; Lack of data for some process model parameters; Complexity of ASMs and ADMs, limiting their application for integration into control schemes; Difficulty in incorporating unmeasurable process disturbances into the process model; Limitation in ADMs in representing some processes involved in AD; Model validation challenges due to substantial data requirements; Limitation to validate some developed models by experimental data; Influence of bioreactor design parameters on the biological reaction; Necessity of studying dynamic hydraulic behaviour alongside mass balances for certain bioreactors; Complexity of fully understanding fouling dynamic in biological membranes and filters; Necessity of investigating SMP and EPS dynamics for MBRs and AnMBRs, in details.	Developed standard mechanistic models by IWA, including ASM1, ASM2, ASM2d, ASM3, ASM4, ASM7, and ADM1, Developed reduced, modified, altered, or simplified ASMs and ADMs, Combined ASMs and ADMs with membrane resistance model for MBR and AnMBR, Employed black-box or AI-based approaches to model the process based on available data.
AOP-based	Non-linearity of the process; Complexity of the process mechanism; Incomplete data due to ROSs instability; Difficulty in sensitivity analysis due to process complexity and lack of data; Limitation of some developed models to steady state operation; Model validation challenges due to substantial data requirements; Influence of reactor design parameters on chemical reactions; Necessity of studying dynamic hydraulic behaviour alongside mass balances for certain reactors.	Employed black-box or AI-based approaches to model processes based on experimental data, Modelled processes using the gray-box approach.

**Table 3:** The studied and proposed control methods in biological and AOP-based wastewater treatment processes.

Type of Control Method	Control Strategy	Note
Linear control	P/PI/PID [40,75,87,88,89,90,91,92,93,94]	*Proportional control (P):* The simplest control strategy; Control action proportional to the error signal; Applicable in on/off and continuous control; Providing stable control within a small-time delay; Oscillatory response with overshoot for large disturbances. *Proportional-Integral control (PI):* Control action based on both current error and cumulative sum of past errors; Integrating integral control for steady-state error elimination; Providing stable control within a moderate time delay; Oscillatory response for high values of integral gain; Slower response than P control. *Proportional-Integral-Derivative Control (PID):* The most implemented control in WWTPs; Control action based on current error, cumulative sum of past errors, and the rate of change in error over time; Integrating derivative control for overshoot elimination; Improved response time; Providing stable control within a larger time delay; Ability to reject more significant disturbances; Complex tuning in some cases; Precise if tuned based on an accurate process model; Low performance for highly nonlinear processes.
	IMC [88,95,96]	Internal model control; A model-based control; Simple design and implementation if process model is achievable; Applicable for a wide range of systems; Control action based on model-based estimation; Beneficial for the processes with a long-time delay; Improper for nonlinear or time-varying systems.
	Pole replacement [97,98,99]	A model-based control; Involving pole replacement to achieve a desired performance; Beneficial for unstable, poorly damped, high order, or large-delayed systems; Improved response by reducing overshoot, settling, and rise time; Complex and knowledge-demanding control method.
	Cascade [12,87,88,100,101,102,103,104,105,106]	A multi-loop control strategy; Adjusting subsequent controller setpoint based on primary controller output; Improved control performance through problem decomposition; Fast and accurate process control performance; Requiring careful consideration of system dynamics; Requiring proper controllers’ tuning.
	FF [107,108]	Early disturbance indication and corrective action generation; Suitable for processes with prolonged delays and frequent disturbances; Applicable when the disturbance is measurable; Enhancing control performance through FB control integration.
	Adaptive [39,79,91,92,99,109]	Self-tuning capability by changing process behaviour; Beneficial for systems with variable dynamics.
	Optimal [81,82]	A model-based control approach; An optimization-based control strategy; Optimized control output; Better performance than conventional controls; Requiring excessive computations; Inefficient for real-time control of overly complex systems.
	MPC [73,79,80,99]	A model-based control approach; Optimization of control actions for short time intervals using process response prediction; Applicable for MIMO systems; Suitable for complex and interdependent processes; Robust control by constraining process variables; Requiring excessive computations; Inefficient for real-time control of overly complex systems.
Nonlinear control	Geometric nonlinear [110,111]	Using invariant sets to capture nonlinear dynamics; Requiring excessive computations; Inefficient for real-time control of overly complex systems.
	Gain scheduling [99,110]	Control of diverse operating regimes by adaptable control gains; Gain scheduling based on process models; Dynamic control by switching between sets of control gains.
	NMPC [12,112]	MPC control for a nonlinear process.
	NMC [113,114,115]	IMC control for a nonlinear process.
AI-based control	Expert system (Knowledge-based) [102,106]	Intelligent-based control systems; Emulating human expert thinking for generating control action.
	Fuzzy logic-based [40,91,106,116]	Implementing FLC or fuzzy-based process models in the structure of other control methods such as MPC or IMC.
	ANN-based [75,76,79,95,96,112,117,118,119,120]	Efficient in capturing the nonlinearity of the system; Used with model-based control approaches; The most common ANNs in wastewater treatment applications: feedforward neural networks (FFNN), RBFNN, and recurrent neural networks (RNN).
	Nature-inspired algorithm-based [121]	Efficient in capturing the nonlinearity of the system; Problem-solving algorithms derived from natural processes such as ANN, GA, PSO, and ant colony optimization (ACO); Integration into control schemes for process modelling or control action optimization.
	Hybrid AI-based [93,122,123,124,125]	Integration of diverse AI-based methods for enhanced benefits and mitigation of individual limitations.

**Table 4:** Recent studies on controlling biological and AOP-based wastewater treatment processes.

Wastewater Treatment Process	Influent Wastewater Data Origin	Reactor Size	Dynamic Process Model	Control Strategy	Control Parameters	Study Focus and Other Information	Reference
ASP	Visakhapatnam MWWTP, India	Full scale: two anoxic tanks, 663 $m^{3}$ each, three aerobic tanks, 883 $m^{3}$ each	ASM1 in BSM1	PI, FPI, MPC, FLC	CVs: ${DO}_{r}$, ${\left[{NO}_{3,}^{-}\right]}_{r}$; MVs: aeration rate, internal recycling flow rate	Simulation; controller tuning methods: iSIMC for PI, a proposed method for FPI	[40]
ASP	Default data for typical MWW data embedded in BSM1	Full scale: two anoxic tanks, three aerobic tanks	BSM1	Data-driven iterative adaptive critic (IAC) control	CVs: DO, ${\left[{NO}_{3,}^{-}\right]}_{r}$; MVs: oxygen transfer coefficient, internal recycling flow rate	Simulation; outperformance of IAC over PID	[109]
ASP	Default data for typical MWW data embedded in BSM2	Full scale	System identification: adaptive fuzzy neural network (AFNN) in BSM2	Data-driven MOPC alongside TMOOA	CVs: DO, ${NO}_{2,r}^{-}$; MVs: oxygen transfer coefficient, internal recycle flow rate	Simulation	[126]
ASP	Făcăi WWTP, Craiova, Romania	Full scale: anoxic tank, 3375 $m^{3}$ and aerobic tank, 15,000 $\;m^{3}$	A modified ASM by Nejjari et al. [127]	Adaptive multivariable control	CVs: DO, inlet wastewater concentration; MVs: aeration rate, RAS rate	Simulation	[39]
ASP	Toulouse City sewer system, France	Pilot scale: a bioreactor, 0.03 $m^{3}$	ASM1	-	-	Simulation; sampling time: 20 min	[41]
ASP	IWW, unknown food industry	Pilot scale: a bioreactor, 0.1 $m^{3}$	System identification: FOPTD-TF by graphical method	Adaptive Gain scheduling	CV: DO; MV: aeration rate	Simulation and implementation; controller tuning method: pole-zero allocation	[99]
ASP	Unknown WWTP	Full scale: one anoxic tank and two aerobic tanks	ASM1 in SIMBA toolbox, MATLAB	Dual QFT loop	CVs: DO, ${\left[{NO}_{3,}^{-}\right]}_{eff}$; MV: aeration rate	Simulation	[128]
ASP	Kartuzy WWTP, Northern Poland	Full scale: four aerobic tanks	ASM2 in SIMBA toolbox, MATLAB	Hierarchical two-level NMPC	CV: DO; MV: aeration rate	Simulation and implementation; sampling time: 5 min	[101]
ASP	LNMIIT WWTP, Jaipur, India	Pilot scale: Capacity of 125 KLD	-	PID implemented in a PLC controller	CV: DO; MV: aeration rate	Simulation and implementation; aeration rate regulation by installing VFD	[129]
ABAC	Nine Springs WWTP, Madison, WI, USA	Pilot scale: five anoxic and aerobic tanks, 2180 L total volume	ASM1 in BSM1	Cascade of PI-P controllers	CV: ${\left[{NH}_{4}^{+}\right]}_{eff}$; MV: aeration rate	Simulation and implementation	[87]
ABAC	Default data for typical MWW data embedded in BSM1		ASM1 in BSM1	Cascade of FLC-PI controllers	CV: DO; MV: aeration rate	Simulation; controller tuning method: IMC-based for PI, sampling time: 15 min	[88]
SBR	Swarzewo WWTP, Poland	Full scale: three anoxic tanks, 5000$\;m^{3}$, three aerobic tanks, 6500 $m^{3}$	ASM2 in SIMBA toolbox, MATLAB	Cascade supervisory sequential controller (SSC)-NMPC	CV: DO; MV: aeration rate	Simulation; sampling time: 2 min	[12]
SBR	Unknown (data is available in the study)	Full scale	ASM2d in MATLAB	Fuzzy control	CV: DO; MV: oxygen transfer coefficient	Simulation	[130]
SBR	Cerlà WWTP, Spain	Pilot scale	-	On/off, PID, fuzzy control implemented in Labwindows^®^	CV: DO; MV: aeration rate	Simulation and implementation; outperformance of fuzzy control	[131]
Oxidation ditch	Yumoto WWTP, Japan	Full scale	ASM3 and ASM2d in WEST	Combination of FB and FF	CV: OR; MV: aeration rate	Simulation and implementation	[108]
MBR	Unknown WW	Pilot scale	FFNN, RBFNN, NARXNN	IMC	CVs: flux, TMP; MV: permeate pump voltage	Simulation	[62]
Series of AD+MBR	Mixture of WW from Palermo WWTP, Italy, and synthetic WW	Pilot scale: anaerobic tank, 62 L, anoxic tank, 102 L, aerobic tank, 211 L, MBR tank, 36 L	Integration of modified ASM2d and physical sub-model	Cascade of PI controllers	CVs: DO, ${\left[{NH}_{4}^{+}\right]}_{eff}$, ${\left[{NO}_{2,}^{-}\right]}_{eff}$; MV: aeration flow rate	Simulation	[103,132]
Up-flow anaerobic fixed bed reactor	Synthetic WW, COD = 8300 mg/L	Lab scale: cylindrical reactor, 1.8 L	-	Rule-based supervisory control	CVs: pH, gas flow rate, methane content; MV: influent flow rate	Simulation and implementation; sampling time: 2.5 min for pH, 30 min for gas flow rate	[102]
Up-flow sludge bed filter	Synthetic ethanol-contained WW representing winery WW	Pilot scale: 1150 L	Modified ADM1	Cascade of PID controllers	CVs: [VFAs]_eff_, Q_methane, eff_; MV: influent flow rate	Simulation; controller tuning method: ISE	[104]
AnMBR	Carraixet WWTP, Valencia, Spain	Full scale: anaerobic tank, 1300 L, two membrane tanks, 800 L each	Resistance-in-series filtration	Hierarchical control, lower layer: PID and on/off, upper layer: fuzzy and rule-based controllers	CVs: fouling rate, TMP, membrane permeability, SRF; MVs: influent flow rate, back-flushing initiation and duration, etc.	Simulation; controller tuning: trial and error for PID, IAE for fuzzy	[106]
UV/H_2_O_2_	Synthetic PVA-contained WW	Lab scale: series of two photoreactors, 0.92 L total volume	System identification: ARX, NARX, HW	FB-PID	CV: effluent pH; MV: ${\left[H_{2}O_{2}\right]}_{in}$	Simulation; controller tuning method: IAE, sampling time: 8 min	[74]
UV/H_2_O_2_	Synthetic PVA-contained WW	Lab scale: series of two photoreactors 0.92 L total volume	System identification: ARX, ARMAX, standard TF, state-space	MPC	CVs: ${\left[TOC\right]}_{eff}$, ${\left[H_{2}O_{2}\right]}_{residual}$; MVs: ${\left[H_{2}O_{2}\right]}_{in}$, feed flow rate; Disturbance: ${\left[PVA\right]}_{feed}$	Simulation; sampling time: 30 s	[73]
Catalytic ozonation	Synthetic WW: paranitrophenol solution, COD = 500 mg/L	Lab scale: reactor, 19 L	Grey-box identification: combining experimental data with mass balance	-	CVs: ${\left[O_{3,gas}\right]}_{outlet}$, UVA_340,eff_; MV: ozonator power	Simulation; sampling time: 8 s	[83]
Ozonation disinfection	Xiangcheng WWTP (XWTP), Suzhou, China	Full scale	System identification: RBFNN trained by PSO	Adaptive MPC	CVs: ozone exposure, ${\left[O_{3,gas}\right]}_{residual}$; MVs: $Q_{O_{3},inlet}$, ${\left[O_{3,gas}\right]}_{inlet}$	Simulation and implementation	[79]
Catalytic ozonation	Synthetic WW: paranitrophenol solution, COD = 500 mg/L	Pilot scale: Reactor, 19 L	System identification TF method, parameter estimation by LM algorithm	Optimal linear quadratic (LQ) control	CVs: ${\left[O_{3,gas}\right]}_{outlet}$; UVA_340,outlet_; MV: ozonator power	Simulation	[81,82]
UV and UV/TiO_2_ disinfection	Miao-Li City sewer system, Taiwan	Lab scale	System identification: BPFNN	Manual control	CV: Total coliform counts in the effluent, MV: $Q_{WW,in}$	Simulation and implementation	[133]
Fenton	Synthetic textile WW (PVA+ Reactive Blue 49 (RB49) and Reactive Black B (RBB) dyes)	Lab scale: initial pH adjusting tank, 0.9 L, main oxidation tank, 1.2 L, second pH adjusting tank, 1.2 L, settling tank, 0.9 L	System identification: BPFNN	ANN-based control	CVs: ${ORP}_{oxidationtank}$, ${pH}_{oxidationtank}$; MVs: $F^{2+}$ dosage, $H_{2}O_{2}$ dosage	Simulation and implementation; sampling time: 30 min	[76]

Notes: MWW: municipal wastewater; IWW: industrial wastewater; MOPC: multi-objective predictive control; TMOOA: transfer multi-objective optimization algorithm; QFT: quantitative feedback theory; LNMIIT: LNM institute of information technology; KLD: kilo liter per day; VFD: variable frequency drive; OR: oxygen requirement; NARXNN: nonlinear autoregressive exogenous neural network; TMP: transmembrane pressure; VFA: volatile fatty acid; SRF: sludge recirculation flow; IAE: integral of absolute error; ARMAX: autoregressive moving average with exogenous input; UVA: UV absorbance; WW: wastewater.

**Table 5:** Motivations and limitations of process control implementation for biological and AOP-based wastewater treatment processes.

Wastewater Treatment Process	Motivations for Implementing Process Control	Limitations
Biological	Essential effluent quality regulations; Necessity of optimizing the removal performance; Necessity of optimizing biogas generation in AD-based processes; Necessity of reducing operating costs; Necessity of decreasing GHGs emission; Variability in influent characteristics/load; Possible changes in environmental regulations; Possibility of emerging new pollutants; Necessity of fouling control in filtration-based processes.	Insufficiently developed process models; Superior effluent quality but limited cost-effectiveness with high aeration in aerobic processes; Limited adjustability range for speed of air compressor or mechanical aerator speed; Restricted aeration adjustment for turbulent maintenance; Limited control in the case of sole focus on DO in aerobic processes; Necessity of controlling multiple parameters to optimize the process; Need for diverse indicators to ascertain optimal SBR phase durations; Interaction of biological and filtration mechanisms in combined processes; Need for real-time data, especially for CVs; Optimal sensor placement.
AOP-based	Essential effluent quality regulations; Necessity of reducing operating costs; Variability in influent characteristics and load; Possible changes in environmental regulations; High possibility of effluent quality alteration by disturbances due to the short time delay.	Insufficiently developed process models; Limited adjustability range for implemented control valves, pumps, and motors; Limited adjustability range for UV dosage in the case of UV-involved processes; Need for real-time data, especially for CVs; The necessity of implementing sensors with short response time due to the short time delay of processes; Toxic effect of some excess materials in the effluent, such as H_2_O_2_; Possibility of formation of complex by-products because of presence of excess reactants in the effluent; Possibility of sluggish response or observing overshoot after implementing controller; High possibility of human error in manual-based control; Optimal sensor placement.

**Table 6:** Recent studies for estimation of some process parameters at WWTPs using data of other measured parameters.

Estimated Parameter	Indicators	Source of Dataset	Modelling Method	Highlights	Reference
${TSS}_{eff}\;{COD}_{eff}\;{TN}_{eff}$	Combination of influent parameters (Q_in_), bioreactor parameters (${DO}_{r}$, ${\left[{NH}_{4}^{+}\right]}_{r},$ ${\left[{NO}_{3}^{-}\right]}_{r}$, ${ALK}_{r}$), effluent parameters (${\left[{NH}_{4}^{+}\right]}_{eff}$, ${ALK}_{eff}$)	Biological treatment unit, unknown WWTP	ANN	Good model fitness for all parameters: ${TSS}_{eff}$: $R^{2}$ = 0.9; ${COD}_{eff}$: $R^{2}$ = 0.88; ${TN}_{eff}$: $R^{2}$ = 0.91	[152]
${TDS}_{eff}\;{BOD}_{5,eff}\;{COD}_{eff}$	${TDS}_{in}$, ${TSS}_{in}$, ${BOD}_{5,in}$, ${COD}_{in}$, ${pH}_{in}$, ${TP}_{in}$ and ${TN}_{in}$	Biological treatment unit, Shokouhieh WWTP, Qom, Iran	Ada Boost Regression (ABR), Gradient Boost Regression (GBR), and Random Forest Regression (RFR)	Outperformance of GBR in predicting target parameters: ${TDS}_{eff}$: CC = 0.962, RMSE = 30.3 mg/L; ${BOD}_{5,eff}$: CC = 0.9, RMSE = 4.6 mg/L; ${COD}_{eff}$: CC = 0.75, RMSE = 9.6 mg/L	[153]
${BOD}_{5,eff}\;{COD}_{eff}\;{TN}_{eff}$ Sludge Volume Index (SVI)	Combination of influent parameters (V_in_, ${COD}_{in}$, ${TSS}_{in}$, ${pH}_{in}$, ${\left[{Cl}^{-}\right]}_{in}$), bioreactor parameters (T, SRT, ${\left[{NH}_{4}^{+}\right]}_{r}$), effluent parameters (${TSS}_{eff}$, ${\left[{NH}_{4}^{+}\right]}_{eff}$, ${pH}_{eff}$, ${\left[{Cl}^{-}\right]}_{eff}$)	Biological treatment unit, Beijing WWTP, China	Multivariate Linear Regression (MLR), Multivariate Relevant Vector Machine (MRVM) and Multivariate Gaussian Processes Regression (MGPR) models	Multi-output soft sensor with good performance	[154]
${COD}_{eff}$	Combination of influent parameters (Q_in_, ${COD}_{in}$, ${BOD}_{5,in}$, ${TSS}_{in}$, ${pH}_{in}$, ${\left[{NH}_{4}^{+}\right]}_{in}$), bioreactor parameters (${DO}_{r}$, ${ORP}_{r}$, RAS flow rate, recycling mixture flow rate)	Biological treatment unit, unknown WWTP	Adaptive estimation: Combination of Hammerstein with wavelet neural networks, adaptive weighted fusion, and approximate linear dependence (ALD) analysis	Outperformance of adaptive model (error% = 6.41)	[155]
${COD}_{eff}\;{TP}_{eff}\;{\left[{NH}_{4}^{+}\right]}_{eff}$	Combination of ${COD}_{in}$, ${TP}_{in}$, ${\left[{NH}_{4}^{+}\right]}_{in}$, reaction time, aeration rate, SRT, MLVSS, filling time, bioreactor parameters (T, SRT, ${\left[{NH}_{4}^{+}\right]}_{r}$)	SBR, Ekbatan WWTP, Tehran, Iran	RBFNN and multi-layer perceptron artificial neural networks (MLPANN)	Good performance of both models; Superior accuracy of MLPANN for all target parameters; Higher R^2^ and lower RMSE in MLPANN for both training and test data	[156]
${TSS}_{eff}\;{BOD}_{5,eff}\;{COD}_{eff}$	Four different combinations: -Only ${TSS}_{in}$, -Only ${BOD}_{5,in}$, -Only ${COD}_{in}$, -All ${TSS}_{in}$, ${BOD}_{5,in}$, and ${COD}_{in}$.	Biological treatment unit; Doha West WWTP	ANN	The best performed input-output models: ${COD}_{in}$-${COD}_{eff}$: R = 0.923, MSE = 0.014; ${COD}_{in}$-${BOD}_{5,eff}$: R = 0.951, MSE = 0.061; ${COD}_{in}$-${TSS}_{eff}$: R = 0.987, MSE = 0.021	[151]

Note: ALK: alkalinity; TDS: total dissolved solids; CC: correlation coefficient; MLVSS: mixed liquor volatile suspended solids; T: Temperature.

## Data Availability

Data are contained within the article.

## Acronyms

ABAC	Ammonia-Based Aeration Control
ABR	Ada Boost Regression
ACO	Ant Colony Optimization
ACR	Anaerobic Contact Reactor
AD	Anaerobic Digestion
ADM	Anaerobic Digestion Model
AE	Aeration Energy
AECI	Aeration Energy Cost Index
AFBR	Anaerobic Fluidized-Bed Reactor
AFNN	Adaptive Fuzzy Neural Network
AFR	Anaerobic Filter Reactor
AI	Artificial Intelligence
ALD	Approximate Linear Dependence
ALK	Alkalinity
AnMBR	Anaerobic Membrane Bioreactor
ANN	Artificial Neural Network
AOP	Advanced Oxidation Process
ARMA	Autoregressive Moving Average Stochastic Model
ARMAX	AutoRegressive Moving Average with eXogenous input
ARX	AutoRegressive with eXogenous input model
AS	Activated Sludge
ASI	Absorbance Slope Index
ASM	Activated Sludge Model
ASP	Activated Sludge Process
BAF	Biological Aerated Filter
BN	Bayesian Network
BOD_5_	5-Days Biochemical Oxygen Demand
BOD_st_	Short-Term Biochemical Oxygen Demand
Boosting-IPW-PLS	Boosting–iterative predictor weighting–partial least square
BPFANN	Backpropagation Function Artificial Neural Network
BSM	Benchmark Simulation Model
CAS	Conventional Activated Sludge
CC	Correlation Coefficient
CDI	Chronic Daily Intake
CNN	Convolutional Neural Network
COD	Chemical Oxygen Demand
Cu-NP	Copper Nanoparticle
CV	Controlled Variable
DAS	Differential Absorbance Spectra
DDM	Data-Driven Model
DenseNet	Densely Connected Convolutional Network
DL	Deep Learning
DO	Dissolved Oxygen
DOM	Dissolved Organic Matter
EB	Electron Beam
EC	Electrical Conductivity
EEM	Excitation–Emission Matrix
EGSB	Expanded Granular Sludge Bed
EPS	Extracellular Polymeric Substance
EQI	Effluent Quality Index
FB	Feedback
FF	Feedforward
FFNN	Feedforward Neural Network
FI	Fluorescence Index
FLC	Fuzzy Logic Control
FOPTD	First-Order Plus Time Delay
FPE	Final Prediction Error
FPI	Fractional order Proportional Integral
FPLS	Fuzzy Partial Least Square
FPLS-DBN	Fuzzy Partial Least Square-Based Dynamic Bayesian Network
GA	Genetic Algorithm
GBR	Gradient Boost Regression
GD	Gradient Descent
GHG	Greenhouse Gas
HGO	High Gain Observer
HQ	Hazard Quotient
HRT	Hydraulic Retention Time
HW	Hammerstein–Wiener Model
IAC	Iterative Adaptive Critic
IAE	Integral of Absolute Error
IFAS	Integrated Fixed Film Activated Sludge
IMC	Internal Model Control
ISE	Integral Square Error
ISE	Ion-Selective Electrode
IWA	International Water Association
IWW	Industrial Wastewater
IWWTP	Industrial Wastewater Treatment Plant
KLD	Kilo Liter per Day
LM	Levenberg–Marquardt algorithm
LNMIIT	LNM Institute of Information Technology
LQ	Linear Quadratic
MATLAB	Matrix Laboratory
MBBR	Moving Bed Biofilm Reactor
MBR	Membrane Bioreactor
MGPR	Multivariate Gaussian Processes Regression
MIMO	Multi-Input Multi-Output
MLFCN	Machine Learning Fully Connected Network
MLPANN	Multi-Layer Perceptron Artificial Neural Network
MLR	Multivariate Linear Regression
MLSS	Mixed Liquor Suspended Solids
MLVSS	Mixed Liquor Volatile Suspended Solids
MOPC	Multi-Objective Predictive Control
MPC	Model Predictive Control
MRVM	Multivariate Relevant Vector Machine
MSE	Mean Squared Error
MV	Manipulated Variable
MWW	Municipal Wastewater
MWWTP	Municipal Wastewater Treatment Plant
NARX	Nonlinear AutoRegressive with eXogenous input model
NARXNN	Nonlinear AutoRegressive eXogenous Neural Network
NMC	Nonlinear Internal Model Control
NMPC	Nonlinear Model Predictive Control
NP	Nanoparticle
NTP	Non-Thermal Plasma
NOM	Natural Organic Matter
O&G	Oil and Grease
OCI	Operational Cost Index
OFPC	Output Feedback Predictive Control
OR	Oxygen Requirement
ORP	Oxidation-Reduction Potential
OUR	Oxygen Uptake Rate
PARAFAC	Parallel Factor Analysis
PDS	Peroxydisulfate
PI	Proportional Integral
PID	Proportional Integral Derivative
PMBC	Process Model-Based Control
PRBS	Pseudo-Random Binary Sequence
PRWWTP	Petroleum Refinery Wastewater Treatment Plant
PSO	Particle Swarm Optimization
PVA	Polyvinyl Alcohol
RAS	Return Activated Sludge
RB49	Reactive Blue 49
RBB	Reactive Black B
RBC	Rotating Biological Contactor
QFT	Quantitative Feedback Theory
RBFNN	Radial Basis Function Neural Network
RFR	Random Forest Regression
RLS	Recursive Least Square
RMSE	Root Mean Square Error
RNN	Recurrent Neural Network
ROS	Reactive Oxygen Species
SAFF	Submerged Aerobic Fixed Film
SBR	Sequencing Batch Reactor
SCADA	Supervisory Control and Data Acquisition
SIMO	Single-Input Multi-Output
SISO	Single-Input Single-Output
SME	Small And Medium Enterprise
SMP	Soluble Microbial Product
SOFNN	Self-Organizing Fuzzy Neural Network
SOPTD	Second-Order Plus Time Delay
SOUR	Specific Oxygen Uptake Rate
SQP	Sequential Quadratic Programming
SRF	Sludge Recirculating Flow
SRT	Sludge Retention Time
SS	Suspended Solids
SSC	Supervisory Sequential Controller
SVI	Sludge Volume Index
SVM	Support Vector Machine
T	Temperature
TDS	Total Dissolved Solids
TF	Transfer Function
TMOOA	Transfer Multi-Objective Optimization Algorithm
TMP	Transmembrane Pressure
TN	Total Nitrogen
TOC	Total Organic Carbon
TP	Total Phosphorous
TPANN	Two-Part ANN
TrOC	Trace Organic Contaminant
TSS	Total Suspended Solids
UASB	Up-flow Anaerobic Sludge Blanket
UI	Unknown Input
UIS	Unknown Input State
UV	Ultraviolet
UVA	Ultraviolet Absorbance
UV-VIS	Ultraviolet–Visible
VFA	Volatile Fatty Acid
VFD	Variable Frequency Drive
WRBFNN	Wavelet Radial Basis Function-Based Neural Network
WWTP	Wastewater Treatment Plant
XWTP	Xiangcheng Drinking Water Treatment Plant
